# Differentiable Formation of Chiroptical Lanthanide Heterometallic Ln_n_Ln’_4‐n_(L_6_) (n=0–4) Tetrahedra with *C*_2_‐Symmetrical Bis(tridentate) Ligands

**DOI:** 10.1002/chem.202201655

**Published:** 2022-08-10

**Authors:** King‐Him Yim, Chi‐Tung Yeung, Melody Yee‐Man Wong, Michael R. Probert, Ga‐Lai Law

**Affiliations:** ^1^ State Key Laboratory of Chemical Biology and Drug Discovery Department of Applied Biology and Chemical Technology The Hong Kong Polytechnic University Hung Hom, Kowloon Hong Kong) China; ^2^ The Hong Kong Polytechnic University Shenzhen Research Institute Shenzhen 518000 P. R. China; ^3^ Chemistry School of Natural and Environmental Sciences Newcastle University Newcastle Upon Tyne NE1 7RU UK

**Keywords:** cage compounds, helicate-to-tetrahedron transformation, lanthanides, supramolecular chemistry

## Abstract

Construction of lanthanide heterometallic complex is important for engineering multifunction molecular containers. However, it remains a challenge because of the similar ionic radii of lanthanides. Herein we attempt to prepare chiral lanthanide heterometallic tetrahedra. Upon crystallization with a mixture of [Eu_2_ **L**_3_] and [Ln_2_ **L**_3_] (Ln=Gd, Tb and Dy) helicates, a mixture of heterometallic Eu_n_Ln’_4‐n_(**L**_6_) (n=0–4) tetrahedra was prepared. Selective formation of heterometallic tetrahedron was observed as MS deconvolution results deviated from statistical results. The formation of heterometallic tetrahedron was found to be sensitive to ionic radii as well as the ratio of the two helicates used in the crystallization.

## Graphical abstract

**Heterometallic supramolecular tetrahedra** can be prepared by self‐assembly of lanthanide ions with ligand **L** followed by crystallization. Upon ether diffusion with a mixture of [Eu_2_ **L**_3_] and [Gd_2_ **L**_3_] helicates, a mixture of heterometallic Eu_n_Gd’_4‐n_(**L**_6_) (n=0–4) tetrahedra was prepared. Ligand **L** demonstrated preferential formation of heterometallic tetrahedron which is confirmed by MS deconvolution.

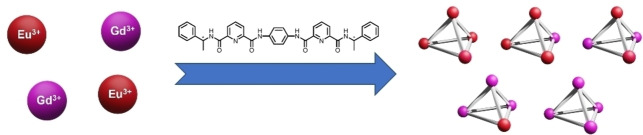

## Introduction

Coordination‐driven supramolecular architecture has received much attention due to various applications such as catalysts,^[1]^ luminescent probes^[2]^ and magnetic materials.^[3]^ A large number of self‐assembled supramolecular edifices such as helicates, cages and metal–organic frameworks have been built based on transition metals.^[4]^ As different types of metal ions have different properties, unique structures and functional role can be tuned by creating a mixed metal system. For instance, in the case of heterometallic supramolecular catalysts, different metal centers can act as either structural nodes to provide a suitable architecture to match the substrate or catalytic centers to interact with target substrates.^[5]^

Lanthanide‐based molecular systems have received immense attention due to their unique physical and chemical properties.^[6]^ A wide variety of d‐f hybrid complex architectures,^[7]^ as well as lanthanide‐based supramolecular systems have been reported.^[8]^ Lanthanide supramolecular systems (mostly monometallic) have been used for luminescent sensing, light‐conversion devices, and as MRI contrasting agents.^[9]^ By combining different lanthanides to form a multi‐functional heterometallic lanthanide system, unique properties for these applications can be enhanced, such as improved up‐conversion efficiency,^[10]^ better optimization optical properties,^[11]^ enhanced catalytic properties, as well as promoting the area in the development of potential dual imaging agents that covers both visible and near‐IR regions.^[12]^

Despite the uniqueness of the f‐elements, lanthanide supramolecular systems are less reported compared with transition metal due to their variable coordination number and poor stereochemical preferences.^[13]^ Hetero‐lanthanide systems are even more challenging due to the similar ionic radii and chemical properties of lanthanide ions.^[14]^ Generally, there are two strategies for constructing hetero‐lanthanide complexes. One of the strategies is to synthesize an organic ligand that consists of different chelating groups that can preferentially bind to different lanthanide ions.^[15]^ However, it is extremely difficult to selectively coordinate different lanthanide ions on target locations within the same ligand. Therefore, the undesired product can be only partially removed from the mixture.^[15b]^ Such complexes are studied as part of a statistical mixture that contains the corresponding fractions of the homometallic species.^[16]^ Another strategy involves a stepwise reaction which performs the complexation sequentially.^[17]^ However, this strategy depends on the use of ligand with strong coordinating power such as a macrocyclic ligand,^[18]^ so that the complexes are stable enough to survive the next steps in the reaction.

To date, only a few hetero‐lanthanide systems have been reported and these are normally low hierarchy structures, i. e. helicate systems, where selectively is achieved by synthetic strategy and ligand design utilising different ligand chelating units to build the structure. Achieving selectivity based on the same chelating units via one pot self‐assembly is considered as impossible because the ligand demonstrates no selectivity towards lanthanide ion. Herein, we demonstrated a selective formation of heterometallic tetrahedron by MS deconvolution.

## Results and Discussion

**Scheme 1:**
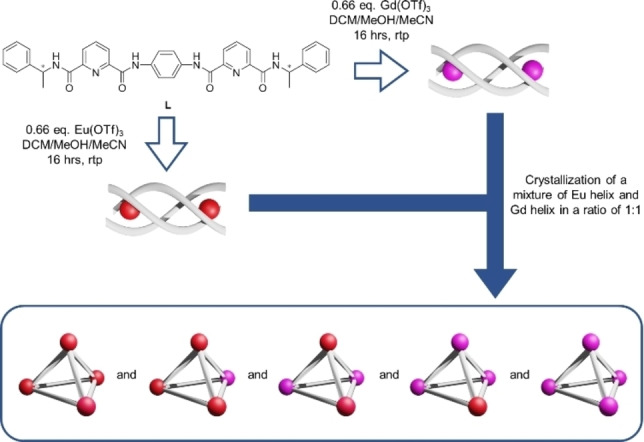
Preparation of mixed heterometallic cage by one‐step self‐assembly followed by ether diffusing crystallization.

Previously we have reported an helicate‐to‐tetrahedron transformation via crystallization with the use of ligand **L**. Such concentration‐dependent supramolecular formation via crystallization has been proven to be a promising way for preparing higher ordered supramolecular complexes by using small building units.[^8d^, ^19^] Ligand **L** was designed based on two chiral metal‐chelating pcam moieties, which were connected with a rod‐like monophenyl linker to form a *C*_2_‐symmetrical bis(tridentate) ligand as reported in our prior work.^[20]^ The corresponding lanthanide complexes were self‐assembled by adding two equivalents of Ln(OTf)_3_ (Ln=La, Sm, Eu, Gd and Lu) into the solution that contained three equivalent of **L** (Scheme 1). This assembly had been shown to undergo helicates to tetrahedra transformation which was dependent on the size of lanthanide cations as well as linker length.^[20]^ Characterizations of these homometallic tetrahedrons are reported in the Supporting Information.

**Figure 1:**
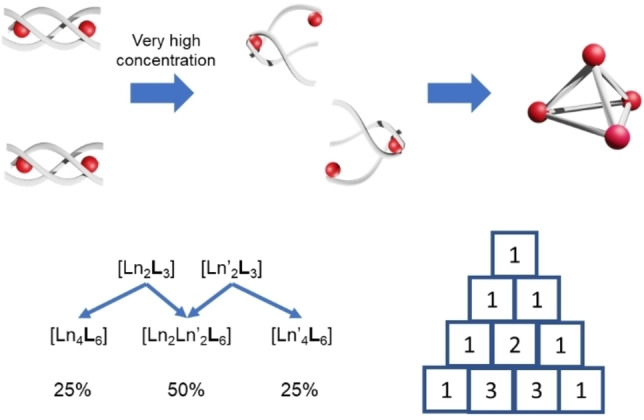
Expected result of helicate‐to‐tetrahedron crystallization upon crystallizing a mixture of [Ln_2_ **L**_3_] and [Ln’_2_ **L**_3_] helicate in a ratio of 1 : 1 based on Pascal's triangle.

With this interesting finding of helicate‐to‐tetrahedron transformation, we further investigated whether a more challenging heterometallic tetrahedron can be designed. For the helicate‐to‐tetrahedron transformation process, it was hypothesized that two helicates would undergo a partial dissociation and then re‐assembled again to form a tetrahedron under high complex concentration. Based on this hypothesis, [Ln_2_ **L**_3_] can associate with either [Ln_2_ **L**_3_] or [Ln’_2_ **L**_3_] to form [Ln_4_ **L**_6_] or [Ln_2_Ln’_2_ **L**_6_] respectively. The ratio of tetrahedra formation was estimated using Pascal's triangle (Figure 1).

**Figure 2:**
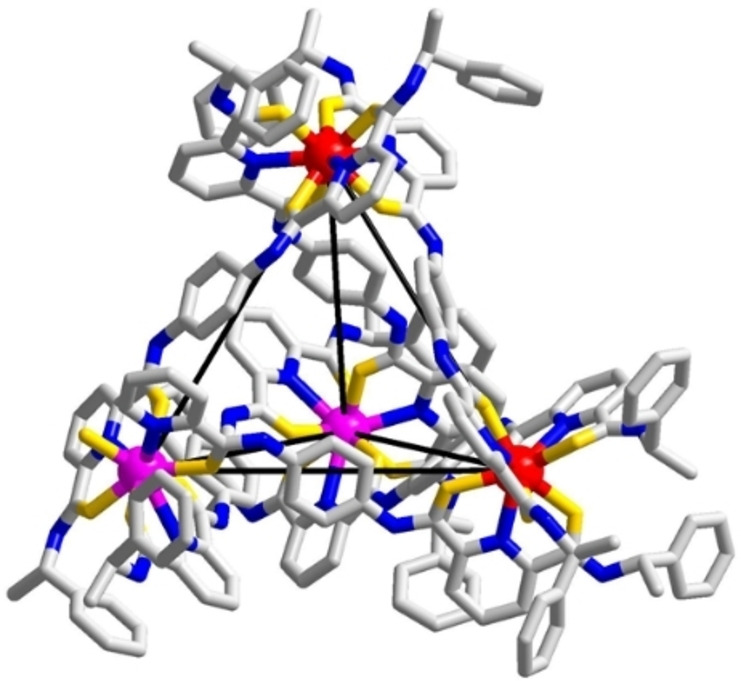
X‐ray crystal structure of [Eu_n_Gd_4‐n_(**L**)_6_] (n=0‐4). The highest species as percentage amount of the sample was found to be [Eu_2_Gd_2_(**L**)_6_] by MS deconvolution. The Eu and Gd metal centers are then refined as 50 : 50 by X‐ray crystallography however depicted as a single element in the illustration.

Upon crystallization of a mixture containing [Eu_2_ **L**_3_] and [Gd_2_ **L**_3_] helicates in a ratio of 1 : 1, a single crystal was obtained via slow ether diffusion. X‐ray crystallographic results revealed a tetrahedral topology between four lanthanide ions and six ligands (Figure 2). Each lanthanide ion could be best described as *D*_3*h*_ tricapped trigonal prism. Average Ln−N (Ln=Eu or Gd) distance was 2.553 Å, which was comparable to an average Eu−N (2.561 Å) and Gd−N (2.581 Å) distance of pyridinedicarboxylic chelating unit. Average Ln−O distance was found to be 2.406 Å, which was also comparable to the average Gd−O and distance Eu−O (2.426 Å and 2.423 Å) reported in literature.^[21]^

**Figure 3:**
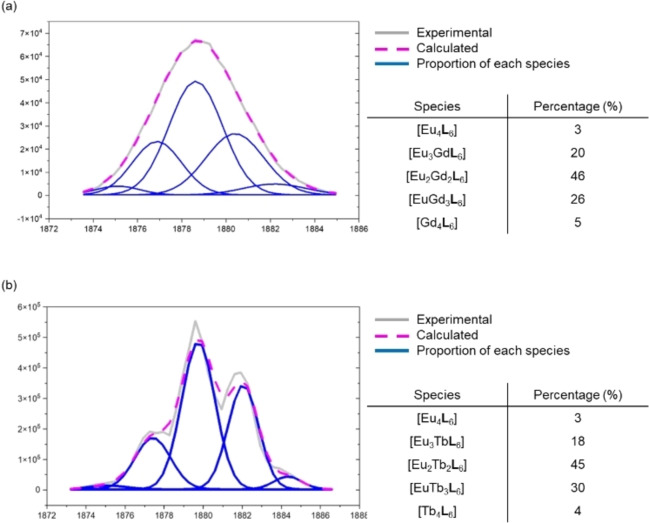
ESI‐HRMS deconvolution of lanthanide tetrahedron crystal based on the intensity of [complex+9OTf^−^]^3+^. (a) MS deconvolution result of [Eu_n_Gd_4‐n_(**L**)_6_] (n=0‐4) (b) MS deconvolution result of [Eu_n_Tb_4‐n_(**L**)_6_] (n=0–4).

Precise differentiation by X‐ray crystallography between Eu and Gd was not possible because of their similar electron density. Characterization by NMR was also not possible either due to the strong paramagnetic nature of Gd. Therefore, the presence of two distinct lanthanide ions was first analyzed by ICP‐OES. ICP‐OES results revealed the presence of both Eu and Gd in the crystallized sample in a ratio of 1 to 1.04 which is similar with the ratio of [Eu_2_ **L**_3_] and [Gd_2_ **L**_3_] used in crystallization. The chemical formula of the isolated tetrahedron was further analyzed by ESI‐HRMS. Because of the similar mass and isotopic pattern of the tetrahedra, MS deconvolution based on peak area was employed.^[22]^ The MS deconvolution result revealed five sets of peak series, which corresponded to a mixture of tetranuclear species [Eu_n_Gd_4‐n_ **L**_6_] (n=0–4) with the progressive loss of anions and protons. The tetranuclear species of the highest percentage amount was found to be [Eu_2_Gd_2_ **L**_6_] (46 %) (Figure 3a). No other homometallic or heterometallic dinuclear species was observed in the ESI‐HRMS (Figure S1). The calculated lanthanide ratio based on the MS deconvolution results (Eu : Gd=1 : 1.03) was also consistent with the results from the ICP‐OES (Eu : Gd=1 : 1.04, Table S1).

By replacing Gd with Tb, a similar result was observed. Upon crystallization of a mixture consisting of [Eu_2_ **L**_3_] and [Tb_2_ **L**_3_] in a ratio of 1 : 1, a mixture of homometallic and heterometallic [Eu_n_Tb_4‐n_ **L**_6_] (n=0–4) tetrahedra was observed as evidenced by MS deconvolution (Figure 3b and S2). Similar to the result of Gd, the highest percentage species was determined to be [Eu_2_Tb_2_ **L**_6_] (45 %). Gd was then further replaced by Dy and a similar result was also observed. From the MS deconvolution result, a mixture of five types of tetrahedra with different proportions of metal content (Eu_4_ **L**_6_], [Eu_3_Dy**L**_6_] [Eu_2_Dy_2_ **L**_6_], [EuDy_3_ **L**_6_] and [Dy_4_ **L**_6_]) was observed (Figures S3 and S4). The highest percentage species in the crystal sample was found to be [Eu_2_Dy_2_ **L**_6_] (46 %).

**Figure 4:**
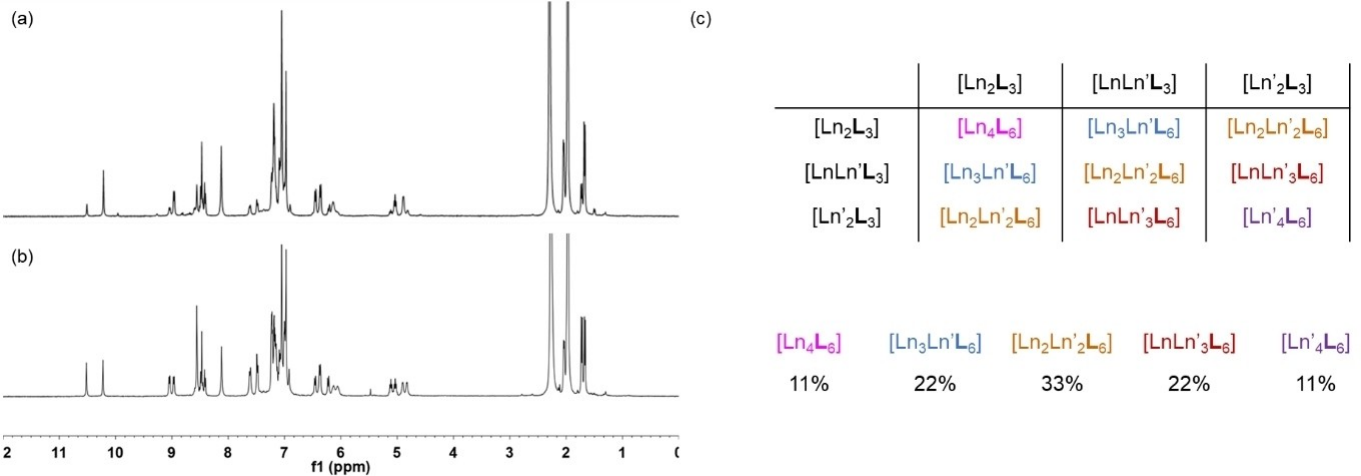
Details of statistical model 1 (M1). ^1^H NMR upon mixing [Eu_2_ **L**_3_] and [Sm_2_ **L**_3_] in a ratio of 1 : 1 in CD_3_CN (a) at t=0 and (b) t=5 hr. (c) Ratio of tetrahedra formation derived from the ^1^H NMR result.

Results showed that the formation of tetrahedron crystal deviated from Pascal's triangle. Pascal's triangle stated that three tetrahedra should be formed in a ratio of 1 : 2 : 1, however we observed the formation of five tetrahedra. In order to study the mechanism of helicate‐to‐tetrahedron transformation, a proton NMR experiment was done for Eu and Sm, which had less paramagnetic nature. Upon mixing [Eu_2_ **L**_3_] and [Sm_2_ **L**_3_] in a ratio of 1 : 1 in CD_3_CN, two helicate underwent inter‐transformation to form a mixture of [Eu_2_ **L**_3_], [EuSm**L**_3_] and [Sm_2_ **L**_3_] in a ratio of 1 : 1 : 1. The system reached an equilibrium after 5 h and did not change upon heating (Figures 4a and 4b). Based on this proton NMR result, a statistical model M1 was proposed (Figure 4c). In M1, five different tetrahedra with different proportions of lanthanide ions were formed, their statistical species is shown in Figure 4c.

Previously, we have reported that the ionic radii will govern the supramolecular formation of **L**.^[20]^ Based on the MS deconvolution result, we hypothesized that lanthanide ions with similar ionic radii would have similar rates of association and dissociation, which caused similar formation in the mixture of [Ln_n_Ln’_4‐n_ **L**_6_] (n=0–4) tetrahedron species. Therefore, further investigation on ionic radii difference was performed by employing early lanthanide (La) and late lanthanide (Lu). In the case of La, the major species were found to be [La_2_ **L**_3_], [EuLa**L**_3_] and [Eu_2_ **L**_3_], and the minor species were found to be [Eu_2_La_2_ **L**_6_] and [Eu_3_La**L**_6_] (Figure S5). Since La has preferential to form helicates in this system, we believed that this caused the bias in the resulting species when the system underwent the helicate‐to‐tetrahedron transformation when La was involved. Therefore, the resulting crystallized product consisted of a mixture of five different species but comprised mostly of the helicate species.

For Lu, five types of tetrahedron were obtained and the highest percentage of the species was found to be [Eu_4_ **L**_6_] (59 %) but not [Eu_2_Lu_2_ **L**_6_] (8 %) (Figure S6 and S7). Upon mixing [Eu_2_ **L**_3_] and [Lu_2_ **L**_3_] in a ratio of 1 : 1 in CD_3_CN, two helicates underwent a slow inter‐transformation and the major species was found to be [Eu_2_ **L**_3_] after 5 h (Figure S8). Therefore, the MS deconvolution result showed that the major species was [Eu_4_ **L**_6_] rather than [Eu_2_Lu_2_ **L**_6_]. We then further postulated that Lu complex was relatively more stable due to the small size of Lu^3+^ which slowed the rate of dissociation. In order to confirm this hypothesis, a mixture of [Eu_2_ **L**_3_], [EuLu**L**_3_] and [Lu_2_ **L**_3_] helicate solution in a ratio of 1 : 1 : 1 was prepared (Figure S9). Upon crystallization, MS deconvolution results showed that the highest amount of tetrahedra species formed were [Eu_3_Lu**L**_6_] and [Eu_2_Lu_2_ **L**_6_] (Figures S10 and S11). This can be explained by the increased amount of Eu‐containing helicate, in which the Eu exhibited a relatively faster rate of dissociation and thus favouring the formation of this tetrahedron species.

**Figure 5:**
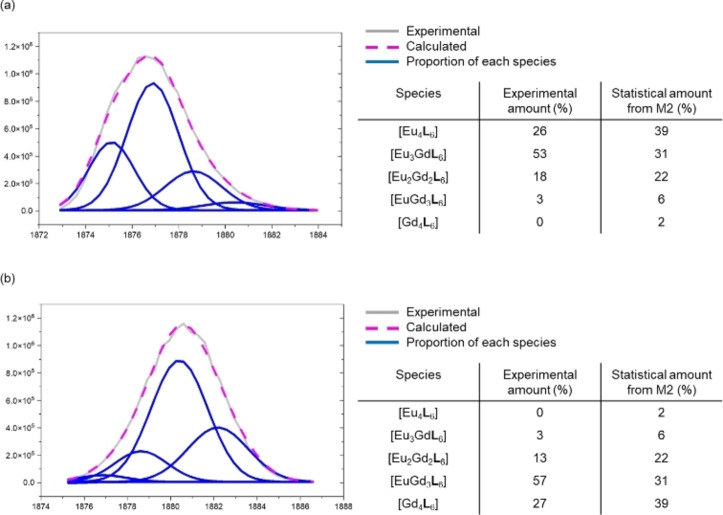
ESI‐HRMS deconvolution of lanthanide tetrahedron crystal based on the intensity of [complex+9OTf^−^]^3+^. (a) MS deconvolution result of [Eu_n_Gd_4‐n_ **L**_6_] (n=0‐4) when Eu : Gd=3 : 1 (b) MS deconvolution result of [Eu_n_Tb_4‐n_ **L**_6_] (n=0–4) when Eu : Gd=1 : 3.

Based on M1, we hypothesized that the formation of [Ln_n_Ln’_4‐n_ **L**_6_] (n=0–4) depended not only on the ionic radii of lanthanide ions, but also on the amount of lanthanide ions that was present in the solution. Thus, a new statistical model M2 was established based on the ^1^H NMR result of a mixture of [Eu_2_ **L**_3_] and [Sm_2_ **L**_3_] in a ratio of 3 : 1 (Figures S12 and S13). The statistical result showed that the highest amount of tetrahedron species should be homometallic [Eu_4_ **L**_6_]. Interestingly, upon crystallization of a mixture containing [Eu_2_ **L**_3_] and [Gd_2_ **L**_3_] helicates in a ratio of 3 : 1, the highest percentage species of tetrahedron was found to be [Eu_3_Gd**L**_6_] (53 %) rather than [Eu_4_ **L**_6_] (Figure 5a). This indicated a slight selectivity towards the formation of heterometallic tetrahedron based on lanthanide ratio. A similar result was also observed when the ratio of [Eu_2_ **L**_3_] and [Gd_2_ **L**_3_] was changed from 3 : 1 to 1 : 3 (Figure 5b). By comparing the experimental result with statistical model M2, **L** demonstrated slight selectivity towards the formation of the desired heterometallic [Ln_2_Ln’_2_ **L**_6_] tetrahedron, as evidenced by a higher amount of [Ln_2_Ln’_2_ **L**_6_] and a lower amount of homometallic [Ln_4_ **L**_6_] and [Ln’_4_ **L**_6_].

The MS deconvolution result was further compared with another successful statistical model developed by Hamacek and Piguet and co‐worker.^[16a]^ From their statistical model, upon complexation of ligand with La(III) and Lu(III) in a ratio of 1 : 1, five different lanthanide tetranuclear complexes were formed. The highest percentage amount (∼38 %) of hetero‐lanthanide complex was found to be Ln_2_Ln’_2_ which is consistent with our result. Our result also demonstrated a slight deviation based on their model (Figure S14, Table S3 and S4).

**Figure 6:**
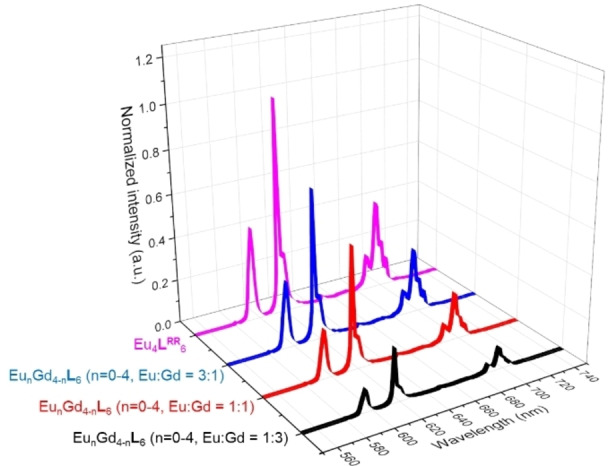
Normalized emission spectrum of [Eu_n_Gd_4‐n_ **L**_6_] (Eu : Gd=1 : 3), [Eu_n_Gd_4‐n_ **L**_6_] (Eu : Gd=1 : 1), [Eu_n_Gd_4‐n_ **L**_6_] (Eu : Gd=3 : 1) and [Eu_4_ **L**_6_] (n=0–4).

**Figure 7:**
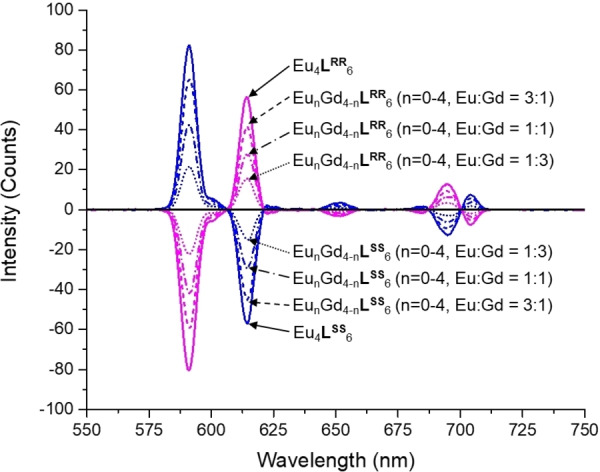
CPL spectrum of [Eu_n_Gd_4‐n_ **L**_6_] (Eu : Gd=1 : 3), [Eu_n_Gd_4‐n_ **L**_6_] (Eu : Gd=1 : 1), [Eu_n_Gd_4‐n_ **L**_6_] (Eu : Gd=3 : 1) and [Eu_4_ **L**_6_] (n=0–4).

The luminescent properties of the mixed heterometallic [Eu_n_Gd_4‐n_ **L**_6_] (n=0–4) tetrahedra were also investigated where successful sensitization of the Eu^III 5^D_0_ excited state via the antenna effect was observed. Upon excitation at 330 nm, characteristic narrow Eu red emission lines that occurred at 595, 616, 688 and 697 nm were observed, which corresponded to the ^5^D_0_ to ^7^F_*J*_ (*J*=1, 2 and 4) transitions (Figure 6). The chiroptical properties of lanthanide [Eu_n_Gd_4‐n_ **L**_6_] (n=0–4) tetrahedra were also further examined to explore the potential of these compounds as chiroptical materials. Solution circular dichroism (CD) measurement was performed in solution state and these five cages were found to have similar CD spectra as expected. Strong cotton effects were observed at 212, 240, 259, 273, 312 and 362 nm and mirror image of CD spectrum were also observed for *R*‐ and *S*‐isomers (Figure S15–S19). [Eu_n_Gd_4‐n_ **L**_6_] (n=0–4) tetrahedra also exhibited interesting circularly polarized luminescence (CPL). The *g*_lum_ (587 nm) value was determined to be 0.15 for [Eu_n_Gd_4‐n_ **L**_6_] (n=0–4). Mirror CPL spectra were also observed for *R*‐ and *S*‐ isomer as expected (Figure 7). The mirror image of both CD and CPL spectra implied successful stereoselective control through point chirality, an observation that was consistent with the results reported by Yeung et al.^[8a]^

## Conclusion

We have successfully demonstrated the selective formation of chiral lanthanide heterometallic [Ln_n_Ln’_4‐n_ **L**_6_] (n=0–4) tetrahedra. With the use of *C*_2_‐symmetrical bis(tridentate) ligand, heterometallic [Ln_n_Ln’_4‐n_ **L**_6_] (n=0–4) tetrahedra can be prepared via crystallization. The construction of heterometallic tetrahedra is highly sensitive to the ionic radii of lanthanide ions. Moreover, **L** also demonstrates a selective formation of heterometallic tetrahedra depending on the ratio of helicates that are involved in the crystallization. Based on the ^1^H NMR study, we further postulate that bimetallic helicate is an essential intermediate for the formation of higher ordered tetrahedron. The heterometallic tetrahedra are found to have CPL signals and serves as promising candidates for the development of chiroptic sensors and materials.

## Experimental Section

**General experimental procedures**: Unless otherwise stated, all chemicals and solvents were obtained commercially and without further purification before used. All moisture‐sensitive compounds were manipulated using standard Schlenk line techniques. All moisture‐sensitive reactions were conducted under a nitrogen atmosphere in glasswares that were oven‐dried at 140 °C overnight prior to use. Anhydrous dimethylformamide (DMF) and N,N‐Diisopropylethylamine (DIPEA) were purchased from Acros. Other solvents were used as received. Grace silica gel 60 (40–63 mesh) was used for column chromatography. 1D NMR spectra were recorded on a Bruker Ultrashield Advance Pro 400 MHz instrument and Jeol ECZ500R 500 MHz NMR spectrometer and the chemical shifts were determined with tetramethylsilane (TMS) or solvents in parts per million (ppm). ESI‐HRMS were performed on a Waters Synapt G2‐Si Quadrupole MS. Elemental analyses were performed on an Elementar Vario EL cube elemental analyzer. ICP‐OES was performed on Agilent 710 Series Inductive Coupled Plasma Optical Emission Spectroscopy for elemental analysis.

Single‐photon luminescence spectra were recorded using an Edinburgh Instrument FLSP920 spectrophotometer that was equipped with Xe900 continuous xenon lamp, μF920 microsecond flashlamp and a single photon counting Photomultiplier Tube. The excitation and emission spectra recorded on the FLSP920 were corrected with the correction file from the F900 software. CD spectra were recorded with a Jasco J‐801 spectropolarimeter with a 1 cm cell at 25 °C. and presented as Δϵ in M^−1^ cm^−1^. Hellma quartz cuvettes (1 mm path length) were employed. All spectra were baselined subtracted with the blank solvent. UV‐Visible absorption spectra were recorded with an Agilent Technologies Cary 8454 spectrophotometer. CPL spectra were recorded with a Jasco CPL‐300 circularly polarized luminescence spectrophotometer with a 1 cm cell at 25 °C.

**(S)‐6‐((1‐phenylethyl)carbamoyl)picolinic acid 1^S^ and (R)‐ 6‐((1‐phenylethyl)carbamoyl)picolinic acid 1^R^**: This synthesis is reported by our group previously.^[8a]^ To a stirred solution of 2,6‐pyridinedicarboxylic acid (5 g, 29.90 mmol, 2.50 equiv.) in anhydrous DMF (70 mL) at room temperature, HATU (4.55 g, 11.90 mmol, 1 equiv.) was added in portions over 10 min under nitrogen. After allowing it to stir for 20 min, (S)‐(−)‐1‐phenylethylamine (1.53 mL, 11.97 mmol, 1 equiv.) was added dropwise and the reaction mixture was allowed to stir for 20 min. DIPEA (5.50 mL, 31.60 mmol, 2.60 equiv.) was then added to the reaction mixture over 5 min and the resulting solution was stirred at room temperature for 14 h. The reaction mixture was then diluted with H_2_O (100 mL) and extracted with DCM (30 mL×3), dried with MgSO_4_, filtered, and concentrated in vacuo. Then dissolved it in water and extracted with EA to remove excess DMF. The resulting residue was purified by recrystallization in EtOAc solvent to give a white solid. **1^S^**: (1.46 g, 5.42 mmol, 45.39 % yield), mp 110.2–117.3 °C. ^1^H NMR (400 MHz, CDCl_3_, 298 K) δ: 1.65 (d, 7.0 Hz, 3H), 5.37 (p, J=7.1 Hz, 1H), 7.20–7.44 (m, 5H), 8.01 (d, J=8.2 Hz, 1H), 8.11 (t, J=7.8 Hz, 1H), 8.36 (dd, J=7.7, 1.2 Hz, 1H), 8.48 (dd, J=7.8, 1.2 Hz, 1H).^13^C NMR (400 MHz, CDCl_3_, 300 K) δ: 21.50, 49.14, 126.25, 126.72, 126.95, 127.48, 128.67, 139.50, 142.63, 145.37, 149.56, 162.48, 164.67. HRMS (ESI): *m/z* calcd. for C_15_H_14_N_2_O_3_Na [**1^S^** +Na]^+^: 293.0897, found 293.0895. The enantiomeric purity was determined with HPLC using AS−H column (Hexane/i‐propanol: 80/20; flow rate 1.0 mL/min) and compared with a racemic mixture according to the elution orders with the retention times, t_S_=7.15 mins and t_R_= 9.62 mins) to be >99.9 % ee. **1^R^** was isolated, following the procedure for **1^S^** with the use of (R)‐(+)‐1‐phenylethylamine instead, in 55.10 % yield (1.78 g, 6.59 mmol): mp 110.3–113.9 °C. ^1^H NMR (400 MHz, CDCl_3_, 298 K) δ: 1.60 (d, J=6.9 Hz, 3H), 5.34 (p, J=7.1 Hz, 1H), 7.18–7.41 (m, 5H), 8.06 (t, J=7.8 Hz, 1H), 8.29–8.38 (m, 2H), 8.44 (dd, J=7.8, 1.2 Hz, 1H). ^13^C NMR (100 MHz, CDCl_3_, 300 K) δ: 21.50, 30.94, 49.12, 126.23, 126.69, 126.95, 127.44, 128.63, 139.48, 142.63, 145.41, 149.52, 162.58, 164.80. HRMS (ESI): *m/z* calcd. for C_15_H_14_N_2_O_3_Na [**1^R^** +Na]^+^: 293.0897, found 293.0896. The enantiomeric purity was determined to be 97.0 % ee.

**N^2^,N^2′^‐(1,4‐phenylene)bis(N^6^‐((*S*)‐1‐phenylethyl)pyridine‐2,6‐dicarboxamide) (L^SS^) and N^2^,N^2′^‐(1,4‐phenylene)bis(N^6^‐((*R*)‐1‐phenylethyl)pyridine‐2,6‐dicarboxamide) (L^RR^)**: To a stirred solution of 1^R^ (0.25 g, 0.92 mmol, 2.2 equiv.) in anhydrous DMF (4 mL) at room temperature, HATU (0.765 g, 2.01 mmol, 4.8 equiv.) was added under nitrogen. After allowing it to stir for 20 min, a 4,4’‐diamino‐p‐terphenyl (0.045 g, 0.417 mmol, 1.0 equiv.) was added and the reaction mixture was allowed to stir for 20 min in dark. DIPEA (0.89 mL, 5.1 mmol, 12.5 equiv.) was then added to the reaction mixture and the resulting solution was stirred at room temperature for 14 h. The reaction mixture was then diluted with H_2_O (10 mL) and extracted with DCM (5×3 mL). After removing the organic volatile under reduced pressure, the residue was then washed with MeCN (10 mL), and fine powder was progressively precipitated out. Then the solid was collected by centrifugation and the desired compound was isolated. (L^RR^): (0.20 g, 0.33 mmol, 70 % yield), ^1^H NMR (400 MHz, (CD_3_)_2_SO, 299 K) δ: 1.67 (d, *J=*8 Hz, 6H), 5.35‐5.45 (m, 2H), 7.24 (t, *J=*8 Hz, 2H), 7.34 (t, *J=*8 Hz, 4H), 7.45 (d, *J=*8 Hz, 4H), 7.70 (s, 4H), 8.07 (t, *J=*8 Hz, 2H), 8.35 (d, *J=*8 Hz, 2H), 8.39 (d, *J=*8 Hz, 2H), 9.27 (d, *J=*8 Hz, 2H). ^13^C NMR (100 MHz, (CD_3_)_2_SO, 300 K) δ: 22.17, 48.81, 122.36, 125.41, 126.52, 127.28, 128.80, 134.71, 140.20, 144.52, 149.14, 149.42, 162.28, 163.39. HRMS (ESI): *m/z* calcd. for C_72_H_64_N_12_O_8_Na [2L^RR^+Na]^+^: 1247.4862, found 1247.4850. (L^SS^) was synthesized, following the procedure for (L^RR^) with the use of 1^S^ instead, in 60 % yield (0.17 g, 0.28 mmol): ^1^H NMR (400 MHz, (CD_3_)_2_SO, 299 K) δ: 1.67 (d, *J=*8 Hz, 6H), 5.35‐5.45 (m, 2H), 7.24 (t, *J=*8 Hz, 2H), 7.34 (t, *J=*8 Hz, 4H), 7.45 (d, *J=*8 Hz, 4H), 7.70 (s, 4H), 8.07 (t, *J=*8 Hz, 2H), 8.35 (d, *J=*8 Hz, 2H), 8.39 (d, *J=*8 Hz, 2H), 9.27 (d, *J=*8 Hz, 2H). ^13^C NMR (100 MHz, (CD_3_)_2_SO, 300 K) δ: 22.15, 48.78, 122.35, 125.39, 125.58, 126.51, 127.26, 128.78, 134.71, 140.19, 144.51, 149.13, 149.41, 162.28, 163.20. HRMS (ESI): *m/z* calcd. for C_72_H_64_N_12_O_8_Na [2L^SS^+Na]^+^: 1247.4862, found 1247.4850.

**General synthetic procedures of [Ln_2_L_3_]**: To a white suspension of **L** (10 mg, 0.016 mmol, 1.5 equiv.) in a mixture of 8.49 mL of DCM/MeOH (12 : 1, *v*/*v*), a solution of Ln(OTf)_3_ (0.011 mmol, 1 equiv.) (Ln=La, Sm, Eu, Gd, Tb and Lu) in 7.83 mL of MeCN was added. The solution was changed to homogeneous colorless solution immediately. The solution was then reacted for 16 h at room temperature. After 16 h, the solvent was removed under reduced pressure to give desired product.

[La_2_ **L^SS^**_3_]: (12.0 mg, 3.99×10^−3^ mmol, 73.3 % yield), ^1^H NMR (400 MHz, CD_3_CN, 299 K): δ 10.32 (s, 3×2H), 9.00 (d, *J*=6.9 Hz, 3×2H), 8.56‐8.35 (m, 3×6H), 7.24‐7.01 (m, 3×14H), 5.11 (p, *J*=7.0 Hz, 3×2H), 1.71 (d, *J*=7.1 Hz, 3×6H). ^13^C NMR (100 MHz, CD_3_CN, 300 K) δ: 167.57, 166.99, 148.75, 148.51, 143.14, 141.70, 133.52, 128.51, 127.66, 126.60, 126.31, 125.93, 123.56, 52.25, 20.61. HRMS (ESI) calcd. for C_112_H_96_F_12_La_2_N_18_O_24_S_4_ [La_2_(**L^SS^)**_3_+4OTf^−^]^2+^: 1355.6841, found 1355.6792.

[La_2_ **L^RR^**_3_]: (13.5 mg, 4.48×10^−3^ mmol, 82.4 % yield), ^1^H NMR (400 MHz, CD_3_CN, 299 K) δ: 10.34 (s, 3×2H), 9.01 (d, *J*=7.0 Hz, 3×2H), 8.61–8.39 (m, 3×6H), 7.25–6.97 (m, 3×14H), 5.11 (p, *J*=7.0 Hz, 3×2H), 1.71 (d, *J*=7.1 Hz, 3×6H). ^13^C NMR (101 MHz, CD_3_CN, 300 K) δ: 167.52, 166.92, 148.69, 148.45, 143.05, 141.64, 133.46, 128.44, 127.59, 126.53, 126.25, 125.86, 123.48, 52.21, 20.55. HRMS (ESI) calcd. for C_111_H_95_F_9_La_2_N_18_O_21_S_3_ [La_2_(**L^RR^**)_3_+3OTf^−^‐H^+^]^2+^: 1280.7042, found 1280.7103.

[Sm_2_ **L^SS^**_3_]: (14.5 mg, 4.78×10^−3^ mmol, 87.9 % yield), ^1^H NMR (400 MHz, CD_3_CN, 299 K) δ: 10.40 (s, 3×2H), 9.11 (d, *J*=6.8 Hz, 3×2H), 8.59‐8.31 (m, 3×6H), 7.18 (dd, *J*=5.2, 1.9 Hz, 3×6H), 7.07 (dd, *J*=6.8, 2.8 Hz, 3×4H), 6.98 (s, 3×4H), 5.08–4.92 (m, 3×2H), 1.66 (d, *J*=7.0 Hz, 3×6H). ^13^C NMR (125 MHz, CD_3_CN, 300 K) δ: 169.49, 168.71, 149.49, 149.22, 143.59, 141.77, 133.34, 128.61, 127.66, 125.87, 125.66, 125.32, 123.32, 52.29, 20.63. HRMS (ESI) calcd. for C_111_H_96_F_9_N_18_O_21_S_3_Sm_2_ [Sm_2_(**L^SS^)**_3_+3OTf^−^]^3+^: 861.8131, found 861.8192.

[Sm_2_ **L^RR^**_3_]: (12.1 mg, 3.99×10^−3^ mmol, 73.3 % yield), ^1^H NMR (495 MHz, CD_3_CN, 299 K) δ: 10.15 (s, 3×2H), 8.89 (s, 3×2H), 8.45‐8.32 (m, 3×6H), 7.13 (s, 3×6H), 7.03 (s, 3×4H), 6.92 (s, 3×4H), 5.01–4.95 (m, 3×2H), 1.72–1.50 (m, 3×6H). ^13^C NMR (125 MHz, CD_3_CN, 300 K) δ: 169.48, 168.71, 149.49, 149.23, 143.59, 141.76, 133.34, 128.61, 127.67, 125.87, 125.66, 125.31, 123.32, 52.28, 20.63. HRMS (ESI) calcd. for C_111_H_96_F_9_N_18_O_21_S_3_Sm_2_ [Sm_2_(**L^RR^**)_3_+3OTf^−^]^3+^: 861.8131, found 861.8160.

[Eu_2_ **L^SS^**_3_]: (14.1 mg, 4.64×10^−3^ mmol, 85.4 % yield), ^1^H NMR (400 MHz, CD_3_CN, 299 K) δ: 8.12 (s, 3×4H), 7.40 (s, 3×2H), 7.20 (t, *J*=7.9 Hz, 3×2H), 7.05 (d, *J*=3.9 Hz, 3×10H), 6.45 (d, *J*=8.0 Hz, 3×2H), 6.36 (d, *J*=7.9 Hz, 3×2H), 6.14 (s, 3×2H), 5.04 (s, 3×2H), 2.04 (d, *J*=6.0 Hz, 3×6H). ^13^C NMR (125 MHz, CD_3_CN, 300 K) δ: 160.24, 155.37, 142.84, 142.47, 141.94, 134.83, 128.50, 127.50, 125.65, 125.29, 91.92, 91.80, 51.70, 21.73. HRMS (ESI) calcd. for C_112_H_96_Eu_2_F_12_N_18_O_24_S_4_ [Eu_2_(**L^SS^)**_3_+4OTf^−^]^2+^: 1369.1985, found 1369.1962.

[Eu_2_ **L^RR^**_3_]: (13.6 mg, 4.48×10^−3^ mmol, 82.3 % yield), ^1^H NMR (400 MHz, CD_3_CN, 299 K) δ: 8.07 (s, 3×4H), 7.13 (d, *J*=15.9 Hz, 3×4H), 6.99 (s, 3×10H), 6.37 (s, 3×2H), 6.28 (d, *J*=8.2 Hz, 3×2H), 6.09 (s, 3×2H), 4.80 (s, 3×2H), 1.99 (s, 3×6H). ^13^C NMR (125 MHz, CD_3_CN, 300 K) δ: 163.43, 160.28, 155.37, 142.83, 142.46, 142.02, 134.81, 128.50, 127.50, 125.66, 125.29, 91.94, 91.80, 51.70, 21.73. HRMS (ESI) calcd. for C_112_H_96_Eu_2_F_12_N_18_O_24_S_4_ [Eu_2_(**L^RR^**)_3_+4OTf^−^]^2+^: 1369.1985, found 1369.1949.

[Gd_2_ **L^SS^**_3_]: (12.5 mg, 4.10×10^−3^ mmol, 75.4 % yield), HRMS (ESI) calcd. for C_111_H_96_F_9_N_18_O_21_S_3_Gd_2_ [Gd_2_(**L^SS^**)_3_+3OTf^−^]^3+^: 866.4836, found 861.4828.

[Gd_2_ **L^RR^**_3_]: (14.3 mg, 4.69×10^−3^ mmol, 86.2 % yield), HRMS (ESI) calcd. for C_111_H_96_F_9_N_18_O_21_S_3_Gd_2_ [Gd_2_(**L^RR^**)_3_+3OTf^−^]^3+^: 866.4836, found 861.4865.

[Tb_2_ **L^SS^**_3_]: (14.7 mg, 4.82×10^−3^ mmol, 88.6 % yield), HRMS (ESI) calcd. for C_109_H_94_F_3_N_18_O_15_STb_2_ [Tb_2_(**L^SS^**)_3_+OTf^−^‐2H^+^]^3+^: 767.5113, found 767.5154.

[Tb_2_ **L^RR^**_3_]: (13.6 mg, 4.46×10^−3^ mmol, 81.9 % yield), HRMS (ESI) calcd. for C_112_H_96_Tb_2_F_12_N_18_O_24_S_4_ [Tb_2_(**L^RR^**)_3_+4OTf^−^]^2+^: 1300.7232, found 1300.7169.

[Dy_2_ **L^SS^**_3_]: (14.2 mg, 4.64×10^−3^ mmol, 85.4 % yield), HRMS (ESI) calcd. for C_112_H_96_Dy_2_F_12_N_18_O_24_S_4_ [Dy_2_(**L^SS^**)_3_+4OTf^−^]^2+^: 1379.7060, found 1379.7075.

[Lu_2_ **L^SS^**_3_]: (14.2 g, 4.61×10^−3^ mmol, 84.7 % yield), ^1^H NMR (400 MHz, CD_3_CN, 299 K) δ: 10.42 (s, 3×2H), 9.02 (d, *J*=6.8 Hz, 3×2H), 8.56–8.33 (m, 3×6H), 7.17 (ddd, *J*=8.9, 5.5, 3.3 Hz, 3×6H), 7.02 (d, *J*=5.0 Hz, 3×8H), 4.97 (p, *J*=6.9 Hz, 3×2H), 1.75–1.58 (m, 3×6H). ^13^C NMR (125 MHz, CD_3_CN, 300 K) δ: 166.82, 166.38, 147.04, 146.66, 143.08, 141.74, 133.26, 128.66, 127.61, 126.31, 125.79, 125.69, 123.07, 52.29, 20.77. HRMS (ESI) calcd. for C_111_H_96_F_9_Lu_2_N_18_O_21_S_3_ [Lu_2_(**L^SS^)**_3_+3OTf^−^]^3+^: 878.1615, found 878.1616.

[Lu_2_ **L^RR^**_3_]: (14.4 g, 4.67×10^−3^ mmol, 85.9 % yield), ^1^H NMR (400 MHz, CD_3_CN, 299 K) δ: 10.31 (s, 3×2H), 8.93 (s, 3×2H), 8.38 (s, 3×6H), 7.13 (s, 3×6H), 6.97 (s, 3×8H), 4.92 (d, *J*=7.5 Hz, 3×2H), 1.61 (d, *J*=7.1 Hz, 3×6H). ^13^C NMR (125 MHz, CD_3_CN, 300 K) δ: 166.41, 165.98, 146.63, 146.33, 142.69, 141.32, 128.26, 127.21, 125.87, 125.43, 125.29, 122.64, 51.86, 20.36. HRMS (ESI) calcd. for C_112_H_96_F_12_Lu_2_N_18_O_24_S_4_ [Lu_2_(**L^RR^)**_3_+4OTf^−^]^2+^: 1391.7185, found 1391.7209.

**General procedures for preparing homometallic tetrahedra**: Ln_2_ **L**_3_ (0.66 mmol) was dissolved in MeCN (0.6 mL) and ether was allowed to slowly diffuse into the solution containing Ln_2_ **L**_3_. The solution was decanted, and the crystal was washed with ether and dried under vacuum to obtain the homometallic tetrahedra crystal.

Eu_4_ **L^SS^**_6_: (1.80 mg, 2.96×10^−4^ mmol, 90.0 % yield), ^1^H NMR (495 MHz, CD_3_CN, 299 K) δ: 9.04 (s, 6×4H), 7.81 (s, 6×2H), 7.31 (s, 6×2H), 6.79 (s, 6×4H), 6.40 (d, *J*=31.8 Hz, 6×8H), 5.88 (s, 6×2H), 5.47 (s, 6×2H), 4.09 (s, 6×2H), 2.34 (s, 6×6H). ^13^C NMR (125 MHz, CD_3_CN, 300 K) δ: 161.73, 156.50, 153.58, 142.91, 136.72, 135.57, 133.80, 127.79, 126.76, 125.52, 124.96, 121.93, 119.36, 92.00, 91.32, 52.29, 22.27. HRMS (ESI): *m/z* calcd. for C_225_H_192_Eu_4_F_27_N_36_O_51_S_9_ [Eu_4_(**L^SS^)**_6_+9 OTf^−^]^3+^: 1875.2490, found 1875.2516.

Eu_4_ **L^RR^**_6_: (1.68 mg, 2.77×10^−4^ mmol, 83.8 % yield), ^1^H NMR (495 MHz, CD_3_CN, 299 K) δ: 9.03 (s, 6×4H), 7.80 (s, 6×2H), 7.33 (s, 6×2H), 6.79 (s, 6×4H), 6.40 (d, *J*=30.9 Hz, 6×8H), 5.89 (s, 6×2H), 5.47 (s, 6×2H), 4.11 (s, 6×2H), 2.33 (s, 6×6H). ^13^C NMR (125 MHz, CD_3_CN, 300 K) δ: 161.81, 156.51, 153.57, 142.91, 136.77, 135.56, 133.85, 128.50, 127.79, 126.76, 125.52, 124.96, 121.96, 119.39, 92.10, 91.38, 52.29, 22.26. HRMS (ESI): *m/z* calcd. for C_225_H_192_Eu_4_F_27_N_36_O_51_S_9_ [Eu_4_(**L^RR^)**_6_+9 OTf^−^]^3+^: 1875.2490, found 1875.2516.

Gd_4_ **L^SS^**_6_: (1.45 mg, 2.38×10^−4^ mmol, 72.1 % yield), HRMS (ESI): *m/z* calcd. for C_224_H_192_F_24_Gd_4_N_36_O_48_S_8_ [Gd_4_(**L^SS^**_6_)+8 OTf^−^]^4+^: 1374.4520, found 1374.4512(100 %).

Gd_4_ **L^RR^**_6_: (1.23 mg, 2.02×10^−4^ mmol, 61.2 % yield), HRMS (ESI): *m/z* calcd. for C_225_H_192_F_27_Gd_4_N_36_O_51_S_9_ [Gd_4_(**L^RR^**_6_)+9 OTf^−^]^4+^: 1882.2535, found 1882.2499(100 %).

**General procedures for preparing heterometallic tetrahedra**: Ln_2_ **L**_3_ and Ln’_2_ **L**_3_ (depends on different mole ratio) were dissolved in MeCN (0.6 mL) and ether was allowed to slowly diffuse into the solution containing a mixture of Ln_2_ **L**_3_ and Ln’_2_ **L**_3_. The solution was decanted, and the crystal was washed with ether and dried under vacuum to obtain the tetrahedra crystal. The chemical formula of the tetrahedra was first confirmed by analyzing the MS peaks with the highest intensity before subjected to MS deconvolution.

**General idea of statistical model M1 and M2**: Both statistical model M1 and M2 are built based on probability. Detailed mechanism of helicate‐to‐tetrahedron via crystallization is still unknown and it is challenging to monitor such transformation process as a state transition occur in the system (solution helicate to crystallized tetrahedra). Therefore, helicate‐to‐tetrahedron transformation via crystallization is regarded as an aggregation of helicate by random combination. For example, in a solution containing Eu_2_ **L**_3_ and Gd_2_ **L**_3_ helicate in a ratio of 1 to 1, helicate rearrangement occur which generate a mixture of [Eu_2_ **L**_3_], [EuGd**L**_3_] and [Gd_2_ **L**_3_] in a ratio of 1 : 1 : 1. In this case, Eu_2_ **L**_3_ helicate are free to aggregate with Gd_2_ **L**_3_, GdEuL_3_ or Eu_2_ **L**_3_, generating Gd_4_ **L**_6_, Eu_3_Gd**L**_6_, EuGd_3_ **L**_6_, Eu_2_Gd_2_ **L**_6_ or Eu_4_ **L**_6_ tetrahedron. In this crystallization process, both Eu_2_ **L**_3_ and Gd_2_ **L**_3_ have the same probability to aggregate with Eu_2_ **L**_3_. The result of tetrahedron formation is initial helicate ratio in helicate rearrangement before crystallization.

**Crystal structures**: Deposition Number(s) 2092789 (for [Eu_n_Gd_n‐1_ **L** ^**SS**^_6_] (n=0‐4. Eu : Gd=1 : 1)) contain(s) the supplementary crystallographic data for this paper. These data are provided free of charge by the joint Cambridge Crystallographic Data Centre and Fachinformationszentrum Karlsruhe Access Structures service.

## Conflict of interest

The authors declare no conflict of interest.

1

## Supporting information

As a service to our authors and readers, this journal provides supporting information supplied by the authors. Such materials are peer reviewed and may be re‐organized for online delivery, but are not copy‐edited or typeset. Technical support issues arising from supporting information (other than missing files) should be addressed to the authors.

Supporting Information

## Data Availability

The data that support the findings of this study are available in the supplementary material of this article.
