# Supplementary material for: Differentiable Formation of Chiroptical Lanthanide Heterometallic LnnLn’4‐n(L6) (n=0–4) Tetrahedra with C 2‐Symmetrical Bis(tridentate) Ligands
Source: Chemistry. 2022 Aug 10;28(56):e202201655. doi: 10.1002/chem.202201655 (PMC9805037; doi:10.1002/chem.202201655)
Supplement: Supplementary file 1 — Supporting Information [file CHEM-28-0-s001.pdf]

Chemistry–A European Journal

Supporting Information

Differentiable Formation of Chiroptical Lanthanide Heterometallic $Ln_nLn'_{4-n}(L_6)$ (n = 0–4) Tetrahedra with C_2-Symmetrical Bis(tridentate) Ligands

King-Him Yim, Chi-Tung Yeung, Melody Yee-Man Wong, Michael R. Probert, and Ga-Lai Law*

Table of Contents

1	Data for result and discussion	p.2-15
2	ESI-HRMS analysis	p.16-19
3	Photophysical measurement	p.20-31
4	X-ray crystallography data	p.32-33
5	References	p.33

Data for result and discussion

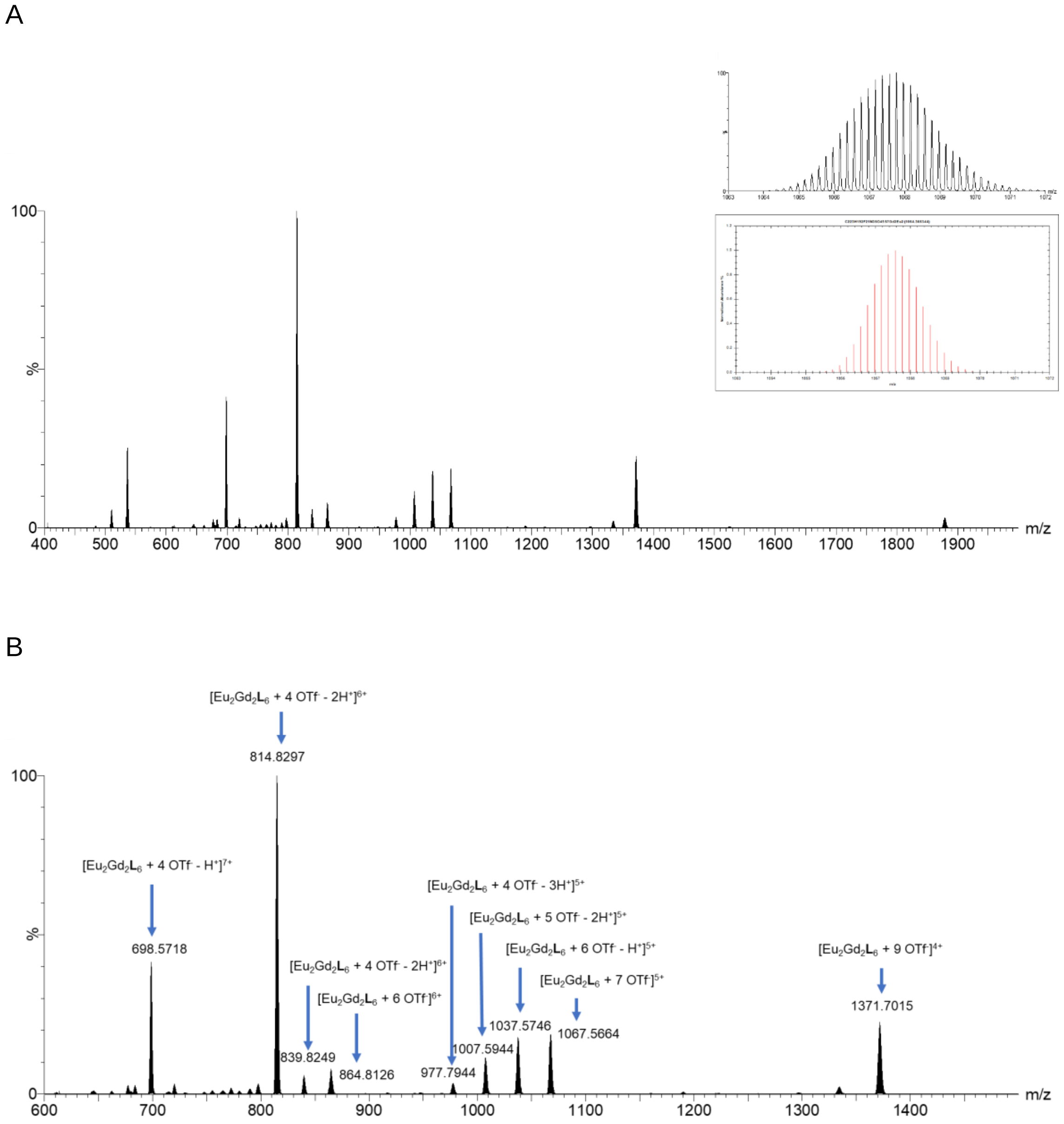

Figure S1. ESI-HRMS of tetrahedra [Eu_nGd_{4-n}**L**$_6$] (n = 0-4, Eu:Gd = 1:1). The chemical formula of the tetrahedra was first confirmed by analyzing the MS peaks with the highest intensity before subjected to MS deconvolution. (A) The full spectrum. Simulated m/z for [Eu_2Gd_2**L**$_6$ + 7 OTf^-]$^{5+}$ is 1067.5697(100%), Experimental found m/z is 1067.5664(100%). Inset showing the experimental (upper) and calculated(lower) isotopic patterns. (B) Expanded region of the mass spectrum to show the possible assignments of the corresponding prominent peaks.

Table S1. ICP-OES result of $Ln_nLn'_{4-n}(\mathbf{L}_6)$ (n=0-4). [a]The concentration was estimated based on figure S10. [b]Negative value was obtained.

Complex	Mass (mg)	Cal Eu (ppm)	Cal Gd (ppm)	Cal Tb (ppm)	Cal Dy (ppm)	Cal Lu (ppm)	Exp Eu (ppm)	Exp Gd (ppm)	Exp Tb (ppm)	Exp Dy (ppm)	Exp Lu (ppm)
$Eu_nGd'_{4-n}(\mathbf{L}_6)$ (n=0-4, Eu:Gd= 1:3)	1.03	2.575	7.983	0	0	0	2.296	6.810	/[b]	0.060	/
$Eu_nGd'_{4-n}(\mathbf{L}_6)$ (n=0-4, Eu:Gd= 2:2)	1.00	4.996	5.170	0	0	0	4.517	4.717	/	0.030	/
$Eu_nGd'_{4-n}(\mathbf{L}_6)$ (n=0-4, Eu:Gd= 3:1)	1.24	9.301	3.212	0	0	0	8.953	3.119	/	0.010	/
$Eu_nTb'_{4-n}(\mathbf{L}_6)$ (n=0-4, Eu:Tb= 2:2)	1.18	5.794	0	6.055	0	0	4.888	0.118	5.520	0.038	/
$Eu_nDy'_{4-n}(\mathbf{L}_6)$ (n=0-4, Eu:Dy= 2:2)	0.99	4.851	0	0	5.28	0	3.059	0.085	0.009	3.017	/
$Eu_nLu'_{4-n}(\mathbf{L}_6)$ (n=0-4)[a]	0.37	2.264	0	0	0	1.628	1.990	/	/	/	1.118

A

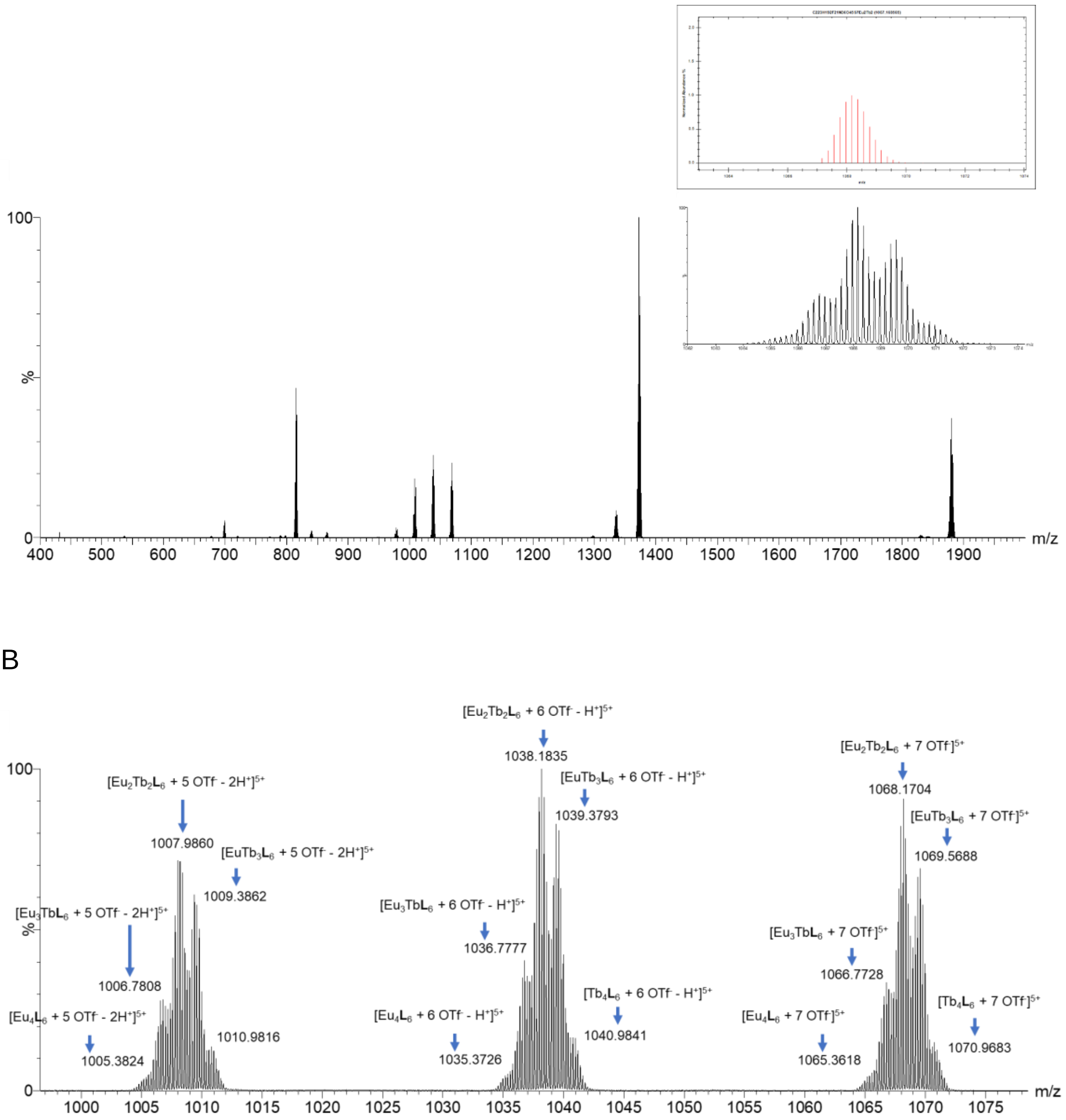

Figure S2. ESI-HRMS of tetrahedra [Eu_nTb_{4-n}**L**$_6$] (n = 0-4, Eu:Tb = 1:1). (A) The full spectrum. Simulated m/z for [Eu_4**L**$_6$ + 7 OTf^-]$^{5+}$ is 1065.3682(100%), [Eu_3Tb**L**$_6$ + 7 OTf^-]$^{5+}$ is 1066.7692(100%), [Eu_2Tb_2**L**$_6$ + 7 OTf^-]$^{5+}$ is 1068.1701(100%), [$EuTb_3$**L**$_6$ + 7 OTf^-]$^{5+}$ is 1069.5711(100%) and [Tb_4**L**$_6$ + 7 OTf^-]$^{5+}$ is 1070.9721(100%), Experimental found m/z for [Eu_4**L**$_6$ + 7 OTf^-]$^{5+}$ is 1065.3618(100%), [Eu_3Tb**L**$_6$ + 7 OTf^-]$^{5+}$ is 1066.7728(100%), [Eu_2Tb_2**L**$_6$ + 7 OTf^-]$^{5+}$ is 1068.1704(100%), [$EuTb_3$**L**$_6$ + 7 OTf^-]$^{5+}$ is 1069.5688(100%) and [Tb_4**L**$_6$ + 7 OTf^-]$^{5+}$ is 1070.9683(100%), Inset showing the experimental (upper) and calculated(lower) isotopic patterns. (B) Expanded region of the mass spectrum to show the possible assignments of the corresponding prominent peaks.

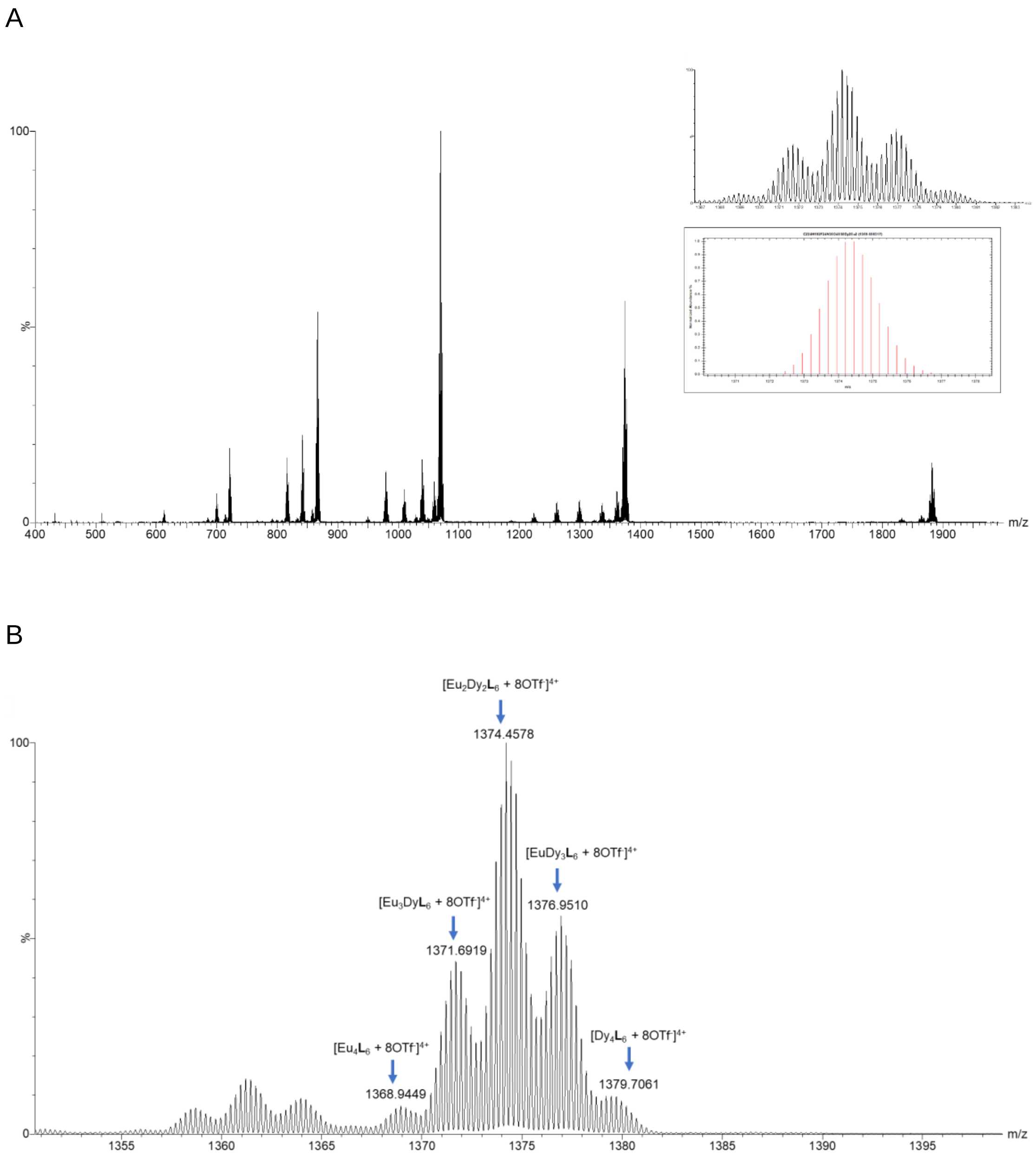

Figure S3. ESI-HRMS of tetrahedra [Eu_nDy_{4-n}**L**$_6$] (n = 0-4, Eu:Dy = 1:1). (A) The full spectrum. Simulated m/z for [Eu_4**L**$_6$ + 8 OTf^-]$^{4+}$ is 1368.9484(100%), [Eu_3Dy**L**$_6$ + 8 OTf^-]$^{4+}$ is 1371.7003(100%), [Eu_2Dy_2**L**$_6$ + 8 OTf^-]$^{4+}$ is 1374.4523(100%), [$EuDy_3$**L**$_6$ + 8 OTf^-]$^{5+}$ is 1376.9540(100%) and [Dy_4**L**$_6$ + 8 OTf^-]$^{4+}$ is 1376.7060(100%), Experimental found m/z for [Eu_4**L**$_6$ + 8 OTf^-]$^{4+}$ is 1368.9449(100%), [Eu_3Dy**L**$_6$ + 8 OTf^-]$^{4+}$ is 1371.6919(100%), [Eu_2Dy_2**L**$_6$ + 8 OTf^-]$^{4+}$ is 1374.4578(100%), [$EuDy_3$**L**$_6$ + 8 OTf^-]$^{4+}$ is 1376.9510(100%) and [Dy_4**L**$_6$ + 8 OTf^-]$^{4+}$ is 1376.7060(100%), Inset showing the experimental (upper) and calculated(lower) isotopic patterns. (B) Expanded region of the mass spectrum to show the possible assignments of the corresponding prominent peaks.

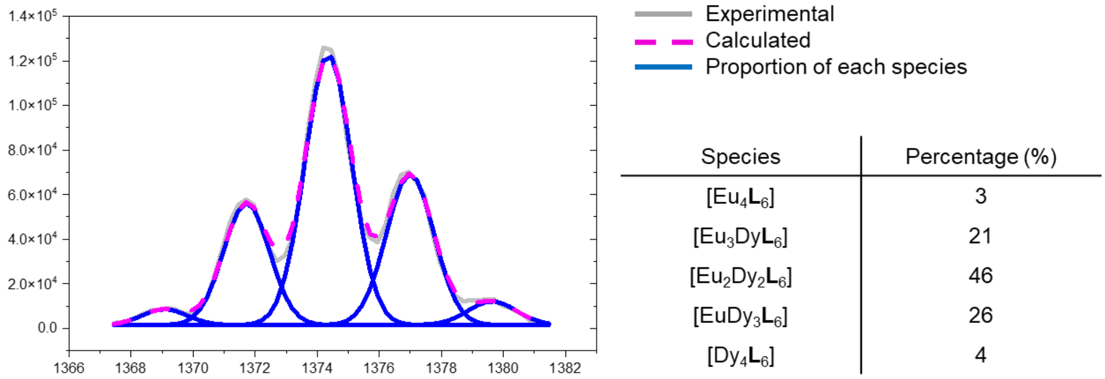

Species	Percentage (%)
[Eu_4**L**$_6$]	3
[Eu_3Dy**L**$_6$]	21
[Eu_2Dy_2**L**$_6$]	46
[$EuDy_3$**L**$_6$]	26
[Dy_4**L**$_6$]	4

Figure S4. ESI-HRMS deconvolution of lanthanide tetrahedron [Eu_nDy_{4-n}(**L**)$_6$] (n = 0-4) crystal based on the intensity of [complex + 8OTf$^-$]$^{4+}$.

Ratio of mixture	Species	Experimental amount (%)	Statistical amount from M1 (%)
1 [Eu_2**L**$_3$] + 1 [Gd_2**L**$_3$]	[Eu_4**L**$_6$]	3	11
	[Eu_3Gd**L**$_6$]	20	22
	[Eu_2Gd_2**L**$_6$]	46	33
	[$EuGd_3$**L**$_6$]	26	22
	[Gd_4**L**$_6$]	5	11
1 [Eu_2**L**$_3$] + 1 [Tb_2**L**$_3$]	[Eu_4**L**$_6$]	3	11
	[Eu_3Tb**L**$_6$]	18	22
	[Eu_2Tb_2**L**$_6$]	45	33
	[$EuTb_3$**L**$_6$]	30	22
	[Tb_4**L**$_6$]	4	11
1 [Eu_2**L**$_3$] + 1 [Dy_2**L**$_3$]	[Eu_4**L**$_6$]	3	11
	[Eu_3Dy**L**$_6$]	21	22
	[Eu_2Dy_2**L**$_6$]	46	33
	[$EuDy_3$**L**$_6$]	26	22
	[Dy_4**L**$_6$]	4	11

Table S2. Comparison of experimental amount and statistical amount of lanthanide tetrahedron. The theoretical peak area of each tetrahedron was first calculated by using Agilent isotopic distribution calculator. MS deconvolution was performed by using the function “peak deconvolution” in OriginPro 2021b. The percentage amount was calculated from the peak area.

A

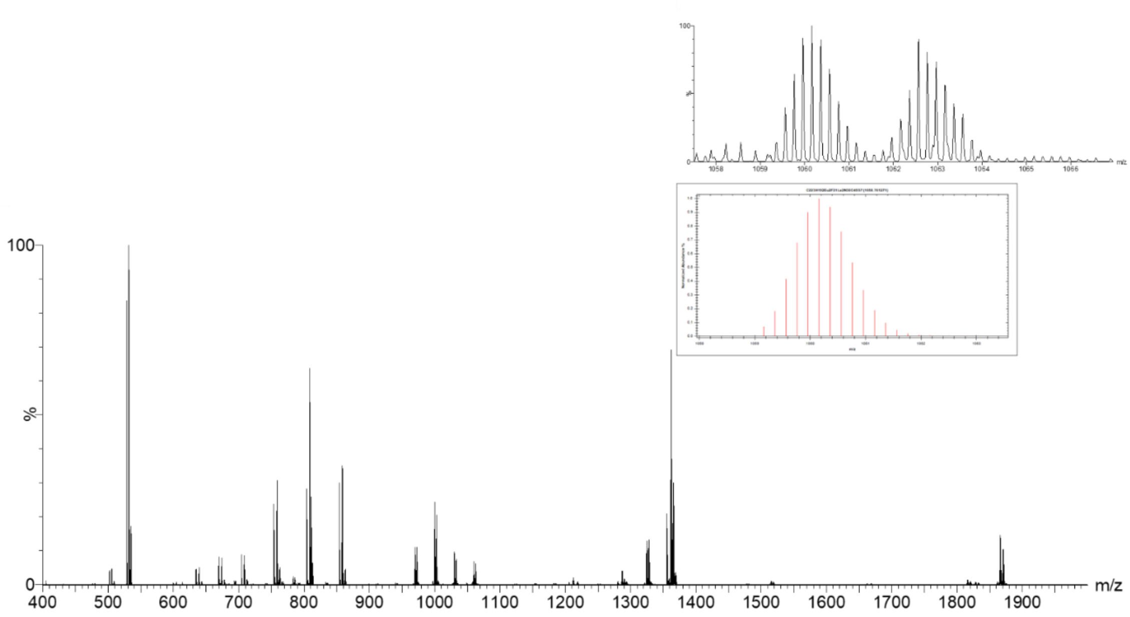

B

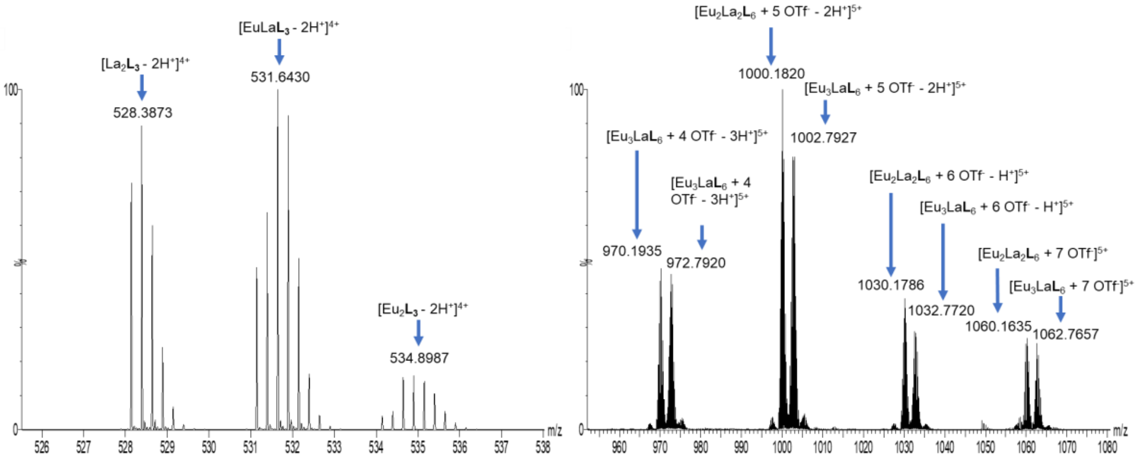

Figure S5. ESI-HRMS of tetrahedron of a mixture of [Eu_2**L**$_3$], [EuLa**L**$_3$], [La_2**L**$_3$], [Eu_2La_2**L**$_6$] and [Eu_3La**L**$_6$] (A) The full spectrum. Simulated m/z for [Eu_2**L**$_3$ - $2H^+]^{4+}$ is 534.8931(100%), [LaEu**L**$_3$ - $2H^+]^{4+}$ is 531.6393(100%), [La_2**L**$_3$ - $2H^+]^{4+}$ is 528.3859(100%), [Eu_2La_2**L**$_6$] + 7 $OTf^-]^{5+}$ is 1060.1625(100%) and [Eu_3La**L**$_6$ + 6 OTf^- - $H^+]^{5+}$ is 1032.7734(100%), Experimental found m/z for [Eu_2**L**$_3$ - $2H^+]^{4+}$ is 534.8987(100%), [LaEu**L**$_3$ - $2H^+]^{4+}$ is 531.6430(100%), [La_2**L**$_3$ - $2H^+]^{4+}$ is 528.3873(100%), [Eu_2La_2**L**$_6$ + 7 $OTf^-]^{5+}$ is 1060.1635(100%) and [Eu_3La**L**$_6$ + 6 OTf^- - $H^+]^{5+}$ is 1032.7720(100%), Inset showing the experimental (upper) and calculated(lower) isotopic patterns. (B) Expanded region of the mass spectrum to show the possible assignments of the corresponding prominent peaks.

A

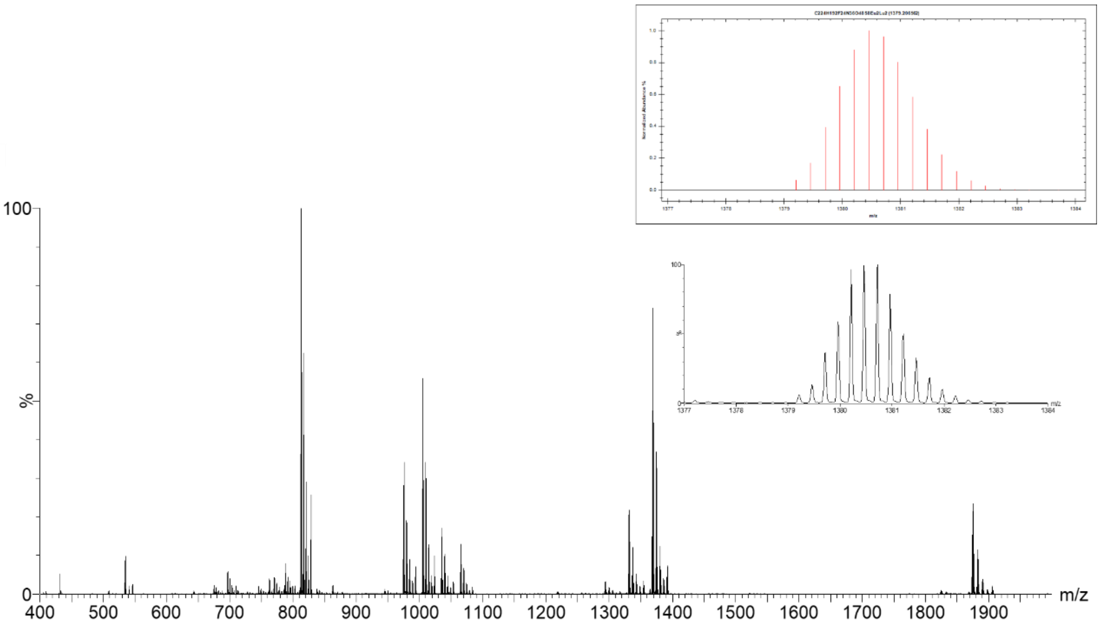

B

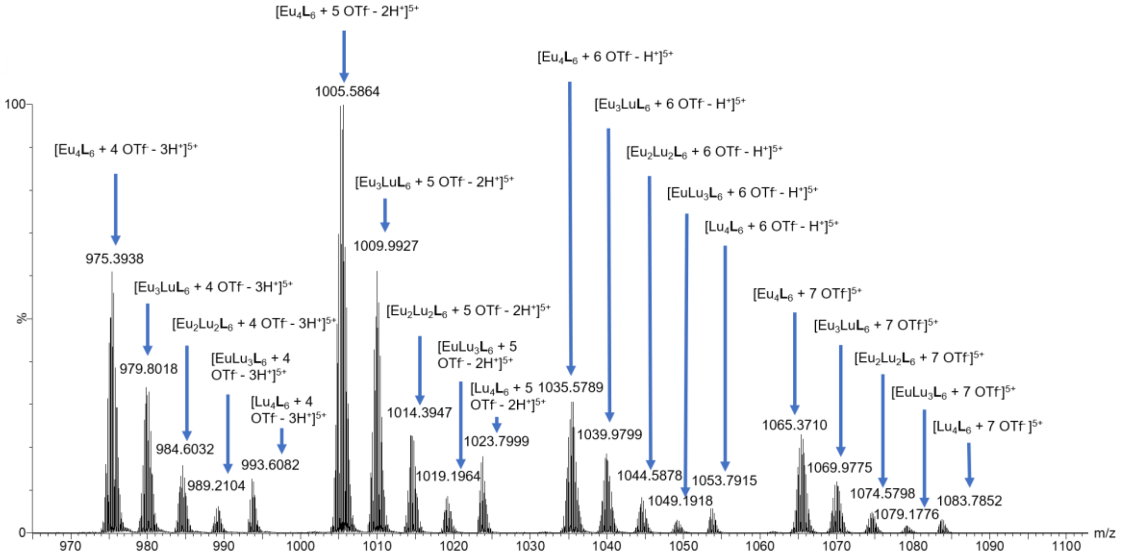

Figure S6. ESI-HRMS of tetrahedron of a mixture of [Eu_nLu_{4-n}**L**$_6$] (n = 0-4, Eu:Lu = 1:1). (A) The full spectrum. Simulated m/z for [Eu_4**L**$_6$ + 8 OTf^-]$^{4+}$ is 1368.9484(100%), [Eu_3Lu**L**$_6$ + 8 OTf^-]$^{4+}$ is 1374.7034(100%), [Eu_2Lu_2**L**$_6$ + 8 OTf^-]$^{4+}$ is 1380.4585(100%), [$EuLu_3$**L**$_6$ + 8 OTf^-]$^{4+}$ is 1386.2136(100%) and [Lu_4**L**$_6$ + 8 OTf^-]$^{4+}$ is 1391.9687(100%), Experimental found m/z for [Eu_4**L**$_6$ + 8 OTf^-]$^{4+}$ is 1368.9503(100%), [Eu_3Lu**L**$_6$ + 8 OTf^-]$^{4+}$ is 1374.7054(100%), [Eu_2Lu_2**L**$_6$ + 8 OTf^-]$^{4+}$ is 1380.4565(100%), [$EuLu_3$**L**$_6$ + 8 OTf^-]$^{4+}$ is 1386.2196(100%) and [Lu_4**L**$_6$ + 8 OTf^-]$^{4+}$ is 1391.9784(100%), Inset showing the experimental (upper) and calculated(lower) isotopic patterns. (B) Expanded region of the mass spectrum to show the possible assignments of the corresponding prominent peaks.

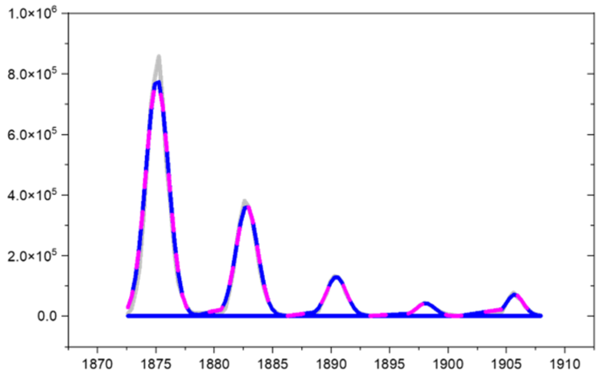

Species	Percentage (%)
[Eu_4**L**$_6$]	59
[Eu_3Lu**L**$_6$]	26
[Eu_2Lu_2**L**$_6$]	8
[$EuLu_3$**L**$_6$]	3
[Lu_4**L**$_6$]	4

Figure S7. ESI-HRMS deconvolution of lanthanide tetrahedron [Eu_nLu_{4-n}(**L**)$_6$] (n = 0-4) crystal based on the intensity of [complex + 9OTf$^-$]$^{3+}$.

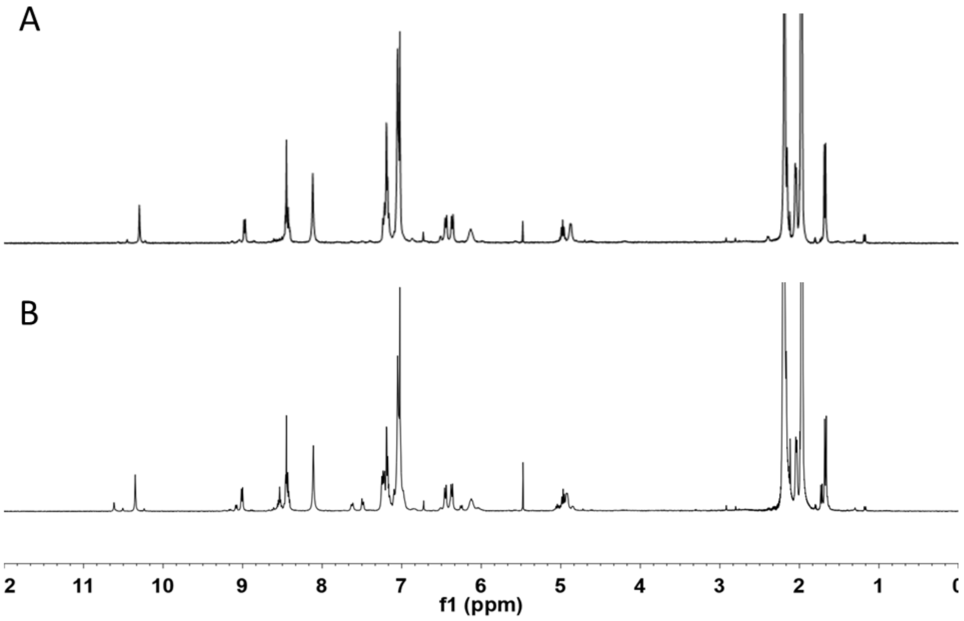

Figure S8. Variation of ^{1}H NMR when [Eu_2**L**$_3$] was mixed with [Lu_2**L**$_3$] after (A) 5 hrs and (B) 7 days in CD_3CN.

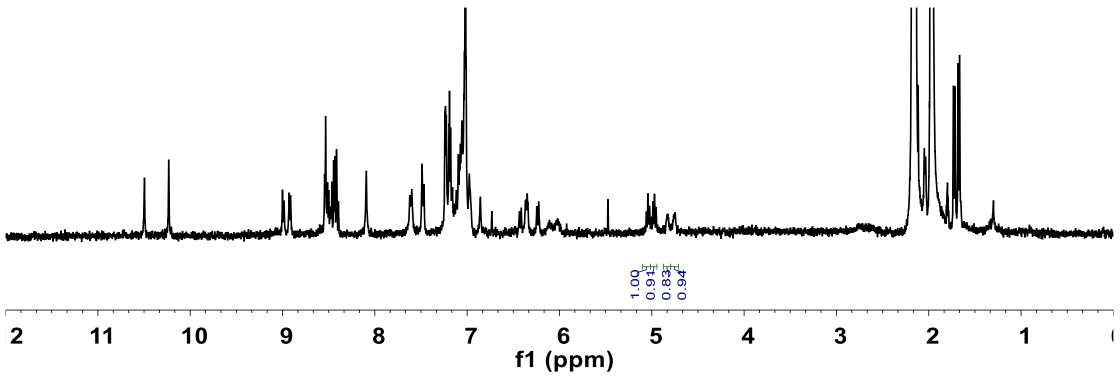

Figure S9. ^{1}H NMR of a mixture of [Eu_2**L**$_3$], [EuLu**L**$_3$], and [Lu_2**L**$_3$] in a ratio of 1:1:1.

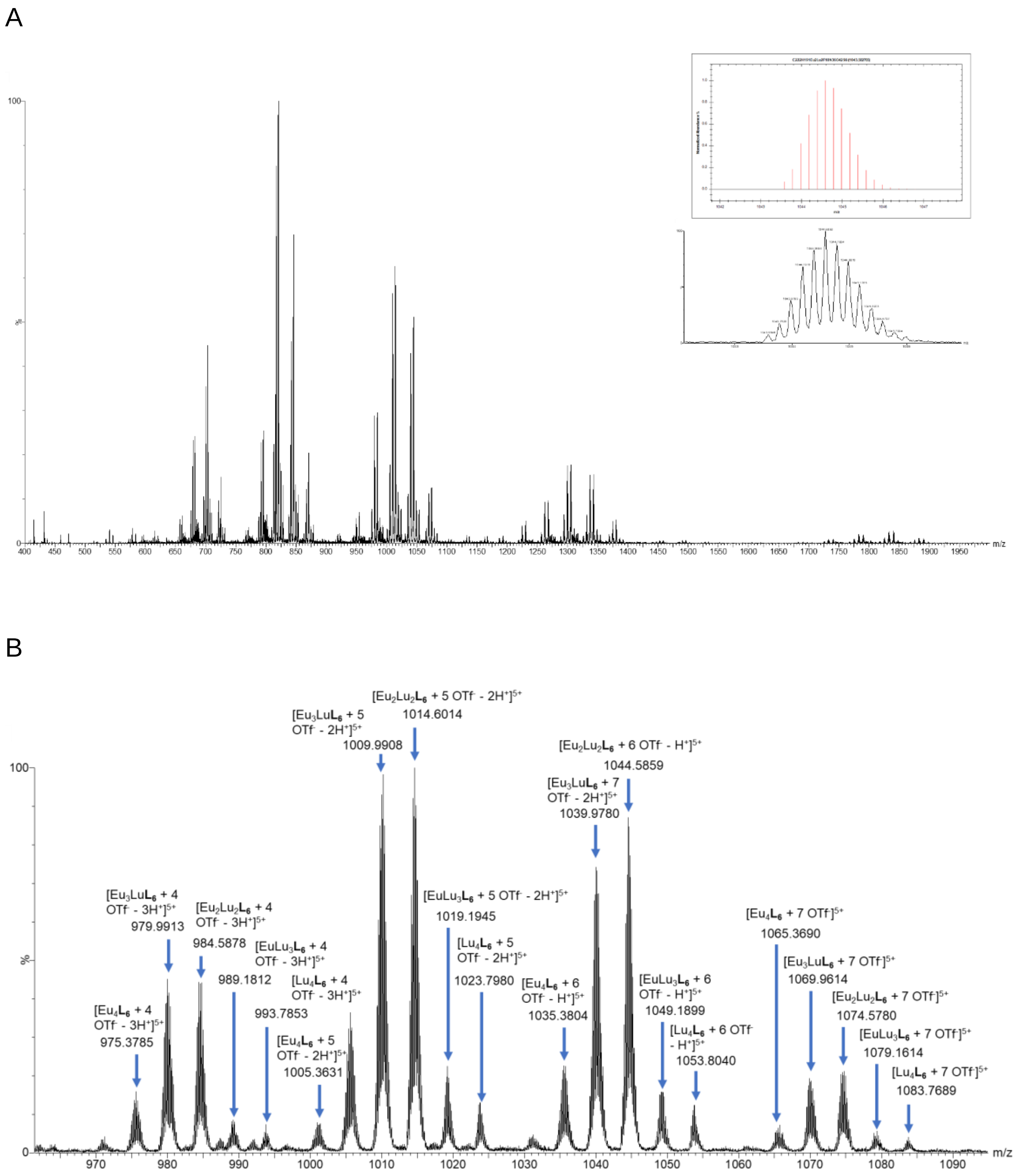

Figure S10. ESI-HRMS of tetrahedron of a mixture of [Eu_nLu_{4-n}**L**$_6$] (n = 0-4, Eu:Lu = 1:1) prepared by crystallization of a mixture of [Eu_2**L**$_3$], [EuLu**L**$_3$] and [Lu_2**L**$_3$] in a ratio of 1:1:1. (A) The full spectrum. Simulated m/z for [Eu_4**L**$_6$ + 6 OTf^- - H^+]$^{5+}$ is 1035.3763(100%), [Eu_3Lu**L**$_6$ + 6 OTf^- - H^+]$^{5+}$ is 1039.9803(100%), [Eu_2Lu_2**L**$_6$ + 6 OTf^- - H^+]$^{5+}$ is 1044.5844(100%), [EuLu_3**L**$_6$ + 6 OTf^- - H^+]$^{5+}$ is 1049.1885(100%) and [Lu_4**L**$_6$ + 6 OTf^- - H^+]$^{5+}$ is 1053.7925(100%), Experimental found m/z for [Eu_4**L**$_6$ + 6 OTf^- - H^+]$^{5+}$ is 1035.3804(100%), [Eu_3Lu**L**$_6$ + 6 OTf^- - H^+]$^{5+}$ is 1039.9780 (100%), [Eu_2Lu_2**L**$_6$ + 6 OTf^- - H^+]$^{5+}$ is 1044.5859(100%), [EuLu_3**L**$_6$ + 6 OTf^- - H^+]$^{5+}$ is 1049.1899(100%) and [Lu_4**L**$_6$ + 6 OTf^- - H^+]$^{5+}$ is 1053.8040(100%), Inset showing the experimental (upper) and calculated(lower) isotopic patterns. (B) Expanded region of the mass spectrum to show the possible assignments of the corresponding prominent peaks.

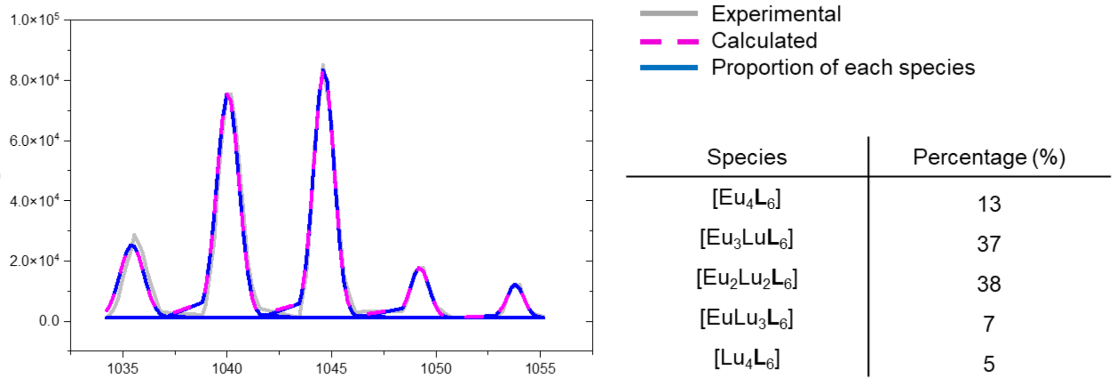

Species	Percentage (%)
[Eu$_4$**L**$_6$]	13
[Eu$_3$Lu**L**$_6$]	37
[Eu$_2$Lu$_2$**L**$_6$]	38
[EuLu$_3$**L**$_6$]	7
[Lu$_4$**L**$_6$]	5

Figure S11. ESI-HRMS deconvolution of lanthanide tetrahedron [Eu$_n$Lu$_{4-n}$(**L**)$_6$] (n = 0-4) crystal based on the intensity of [complex + 6 OTf$^-$ - H$^+$]$^{5+}$.

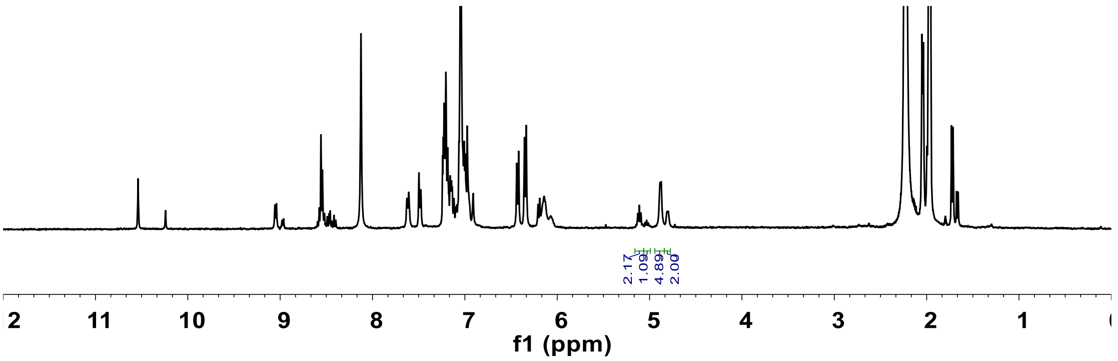

Figure S12. ^{1}H NMR showing a mixture of [Eu$_2$**L**$_3$], [EuSm**L**$_3$] and [Sm$_2$**L**$_3$] in a ratio of 5:2:1 when 3 [Eu$_2$**L**$_3$] was mixed with 1 [Sm$_2$**L**$_3$] after 5 hrs in CD$_3$CN.

	[Ln$_2$**L**$_3$]	[Ln$_2$**L**$_3$]	[Ln$_2$**L**$_3$]	[Ln$_2$**L**$_3$]	[Ln$_2$**L**$_3$]	[LnLn'**L**$_3$]	[LnLn'**L**$_3$]	[Ln'$_2$**L**$_3$]
[Ln$_2$**L**$_3$]	[Ln$_4$**L**$_6$]	[Ln$_4$**L**$_6$]	[Ln$_4$**L**$_6$]	[Ln$_4$**L**$_6$]	[Ln$_4$**L**$_6$]	[Ln$_3$Ln'**L**$_6$]	[Ln$_3$Ln'**L**$_6$]	[Ln$_2$Ln'$_2$**L**$_6$]
[Ln$_2$**L**$_3$]	[Ln$_4$**L**$_6$]	[Ln$_4$**L**$_6$]	[Ln$_4$**L**$_6$]	[Ln$_4$**L**$_6$]	[Ln$_4$**L**$_6$]	[Ln$_3$Ln'**L**$_6$]	[Ln$_3$Ln'**L**$_6$]	[Ln$_2$Ln'$_2$**L**$_6$]
[Ln$_2$**L**$_3$]	[Ln$_4$**L**$_6$]	[Ln$_4$**L**$_6$]	[Ln$_4$**L**$_6$]	[Ln$_4$**L**$_6$]	[Ln$_4$**L**$_6$]	[Ln$_3$Ln'**L**$_6$]	[Ln$_3$Ln'**L**$_6$]	[Ln$_2$Ln'$_2$**L**$_6$]
[Ln$_2$**L**$_3$]	[Ln$_4$**L**$_6$]	[Ln$_4$**L**$_6$]	[Ln$_4$**L**$_6$]	[Ln$_4$**L**$_6$]	[Ln$_4$**L**$_6$]	[Ln$_3$Ln'**L**$_6$]	[Ln$_3$Ln'**L**$_6$]	[Ln$_2$Ln'$_2$**L**$_6$]
[Ln$_2$**L**$_3$]	[Ln$_4$**L**$_6$]	[Ln$_4$**L**$_6$]	[Ln$_4$**L**$_6$]	[Ln$_4$**L**$_6$]	[Ln$_4$**L**$_6$]	[Ln$_3$Ln'**L**$_6$]	[Ln$_3$Ln'**L**$_6$]	[Ln$_2$Ln'$_2$**L**$_6$]
[LnLn'**L**$_3$]	[Ln$_3$Ln'**L**$_6$]	[Ln$_3$Ln'**L**$_6$]	[Ln$_3$Ln'**L**$_6$]	[Ln$_3$Ln'**L**$_6$]	[Ln$_3$Ln'**L**$_6$]	[Ln$_2$Ln'$_2$**L**$_6$]	[Ln$_2$Ln'$_2$**L**$_6$]	[LnLn'$_3$**L**$_6$]
[LnLn'**L**$_3$]	[Ln$_3$Ln'**L**$_6$]	[Ln$_3$Ln'**L**$_6$]	[Ln$_3$Ln'**L**$_6$]	[Ln$_3$Ln'**L**$_6$]	[Ln$_3$Ln'**L**$_6$]	[Ln$_2$Ln'$_2$**L**$_6$]	[Ln$_2$Ln'$_2$**L**$_6$]	[LnLn'$_3$**L**$_6$]
[Ln'$_2$**L**$_3$]	[Ln$_2$Ln'$_2$**L**$_6$]	[Ln$_2$Ln'$_2$**L**$_6$]	[Ln$_2$Ln'$_2$**L**$_6$]	[Ln$_2$Ln'$_2$**L**$_6$]	[Ln$_2$Ln'$_2$**L**$_6$]	[LnLn'$_3$**L**$_6$]	[LnLn'$_3$**L**$_6$]	[Ln'$_4$**L**$_6$]

[Ln$_4$**L**$_6$]	[Ln$_3$Ln'**L**$_6$]	[Ln$_2$Ln'$_2$**L**$_6$]	[LnLn'$_3$**L**$_6$]	[Ln'$_4$**L**$_6$]
39%	31%	22%	6%	2%

Figure S13. Statistical model M2 showing the estimation of resulting tetrahedra. The result was calculated based on the ^{1}H NMR from figure S12.

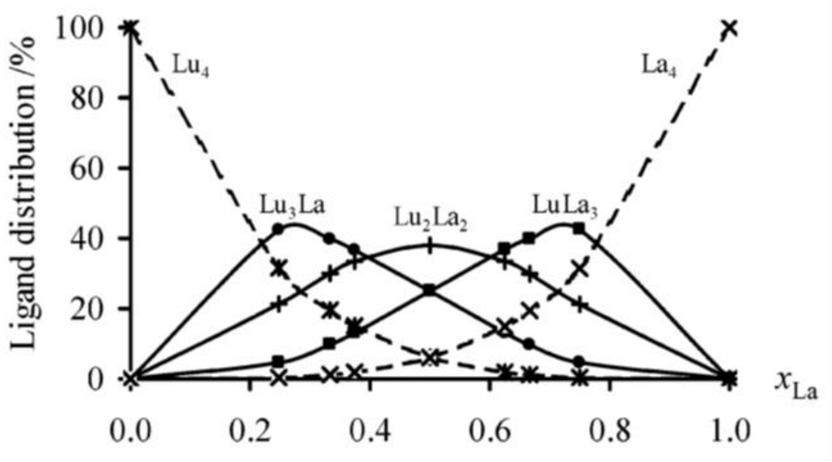

Figure S14. Predicted ligand distributions in the hetero-lanthanide microspecies during titration of ligand with La(III) and Lu(III) reported by Hamacek, Piguet and co-worker.[1]

	Hamacek and Piguet model	MS deconvolution result	Hamacek and Piguet model	MS deconvolution result
	Eu:Gd 1:1	Eu:Gd 1:1	Eu:Gd 1:3	Eu:Gd 1:3
Eu_4	5	3	0	0
Eu_3Gd	25	20	4	3
Eu_2Gd_2	38	46	22	13
$EuGd_3$	25	26	43	57
Gd_4	5	5	31	27

Table S3. Comparison of Eu/Gd complex distribution between Hamacek and Piguet model and MS deconvolution result.

	Hamacek and Piguet model	MS deconvolution result
	Eu:Lu 1:1	Eu:Lu 1:1
Eu_4	5	59
Eu_3Lu	25	26
Eu_2Lu_2	38	8
$EuLu_3$	25	3
Lu_4	5	4

Table S4. Comparison of Eu/Lu complex distribution between Hamacek and Piguet model and MS deconvolution result.

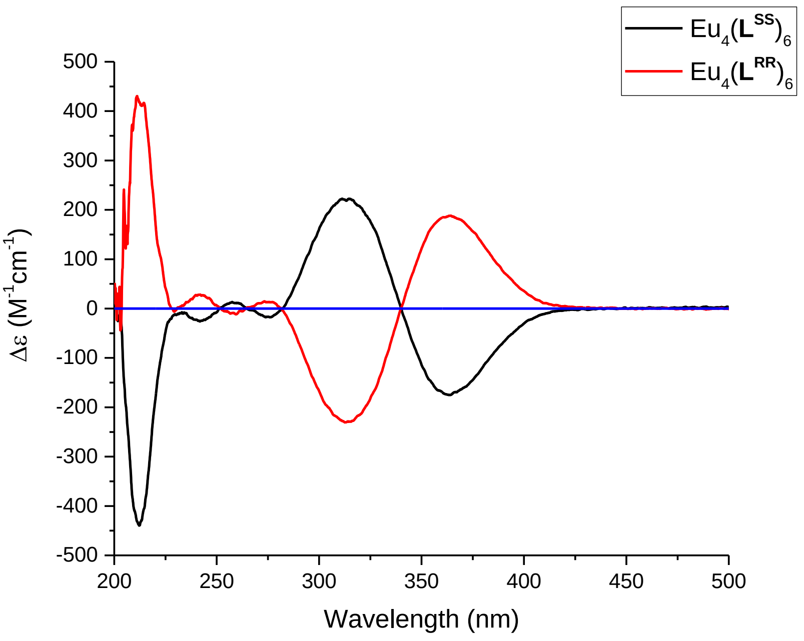

Figure S15. CD spectra of [Eu_4**L**$^{SS}{}_6$]$(OTf)_{12}$ and [Eu_4**L**$^{RR}{}_6$]$(OTf)_{12}$ in MeCN.

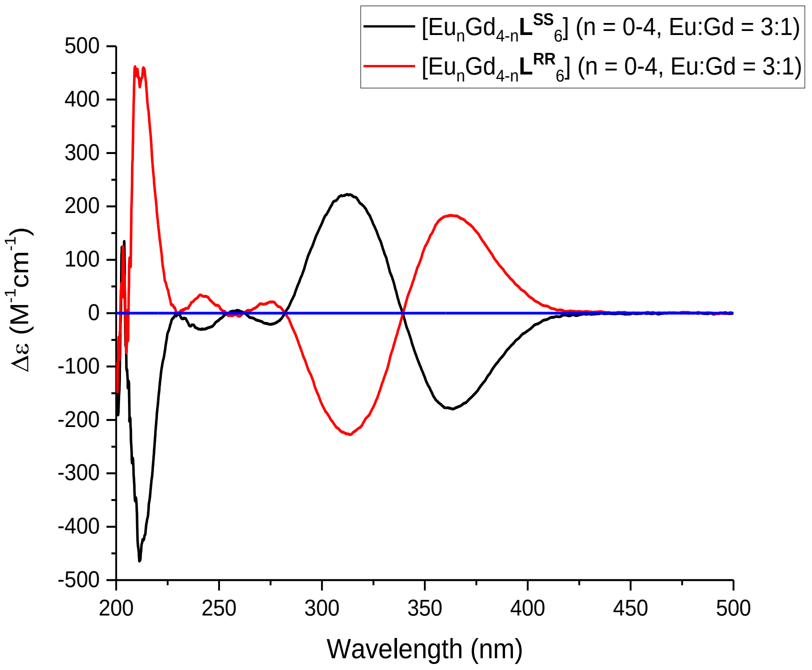

Figure S16. CD spectra of [Eu_nGd_{4-n}**L**$_6$] (n = 0-4, Eu:Gd = 3:1) in MeCN.

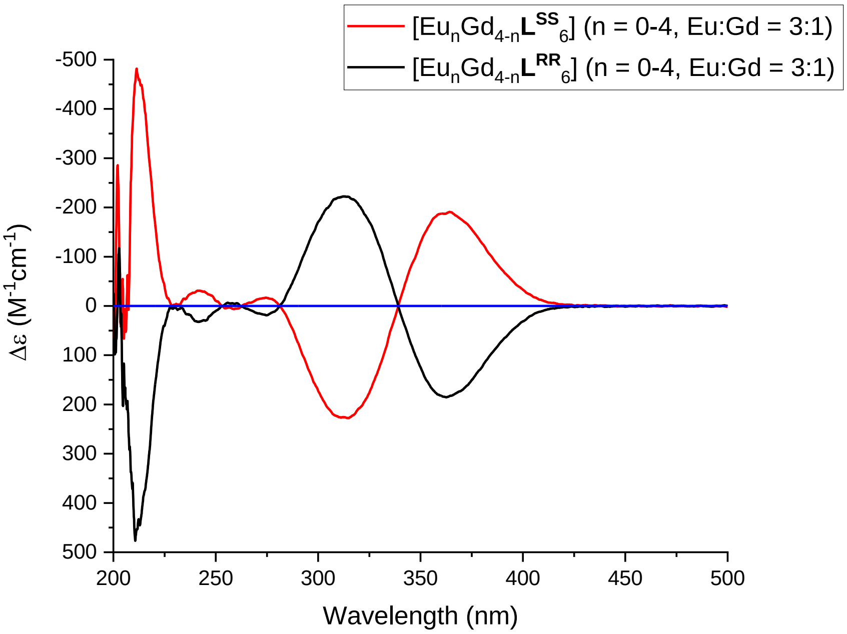

Figure S17. CD spectra of [Eu_nGd_{4-n}**L**$_6$] (n = 0-4, Eu:Gd = 1:1) in MeCN.

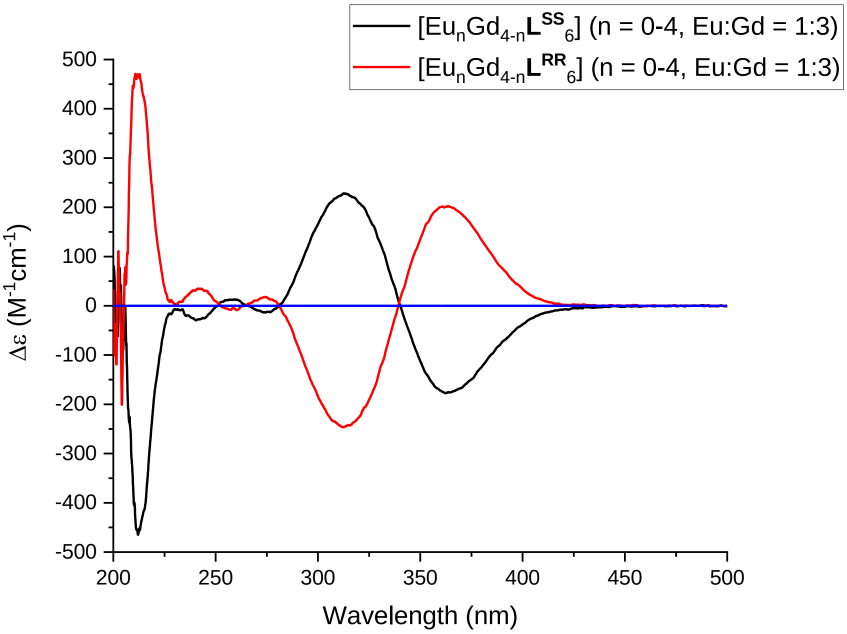

Figure S18. CD spectra of [Eu_nGd_{4-n}**L**$_6$] (n = 0-4, Eu:Gd = 1:3)in MeCN.

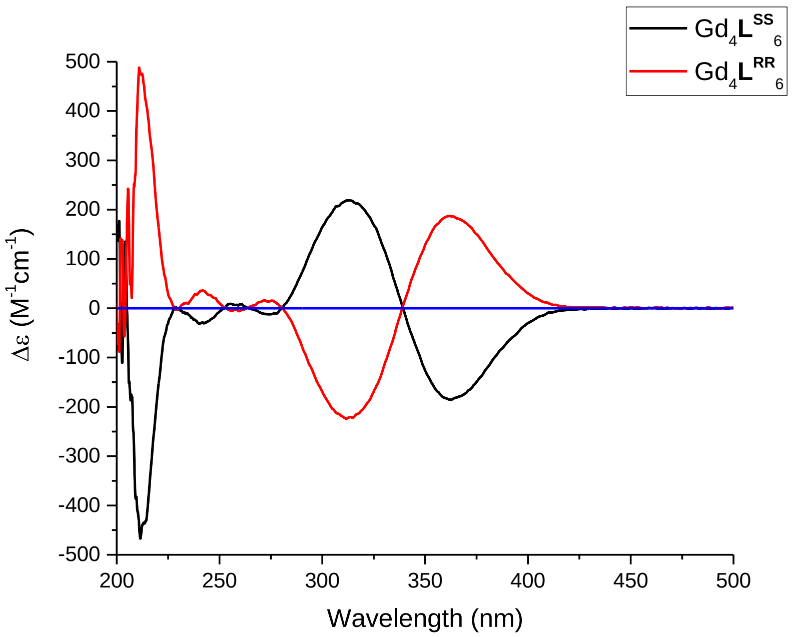

Figure S19. CD spectra of [Gd_4**L**$_6$] in MeCN.

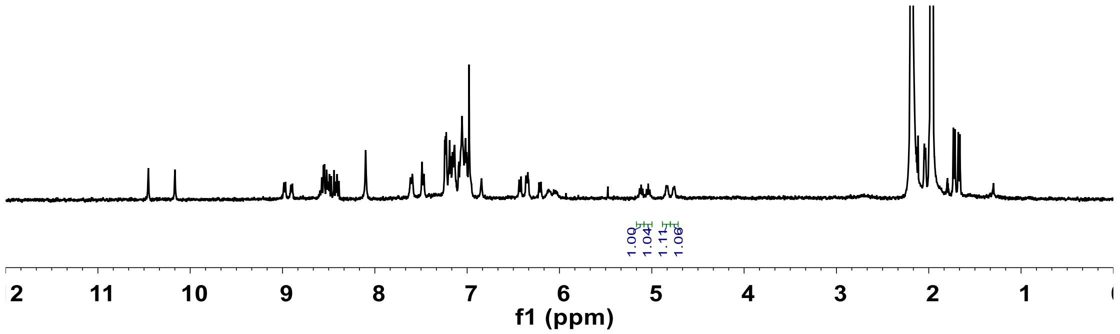

Figure S20. ^{1}H NMR of a mixture of [Eu_2**L**$_3$], [EuSm**L**$_3$], and [Sm_2**L**$_3$] by reacting **L** with $Eu(OTf)_3$ and $Sm(OTf)_3$.

ESI-HRMS analysis

A

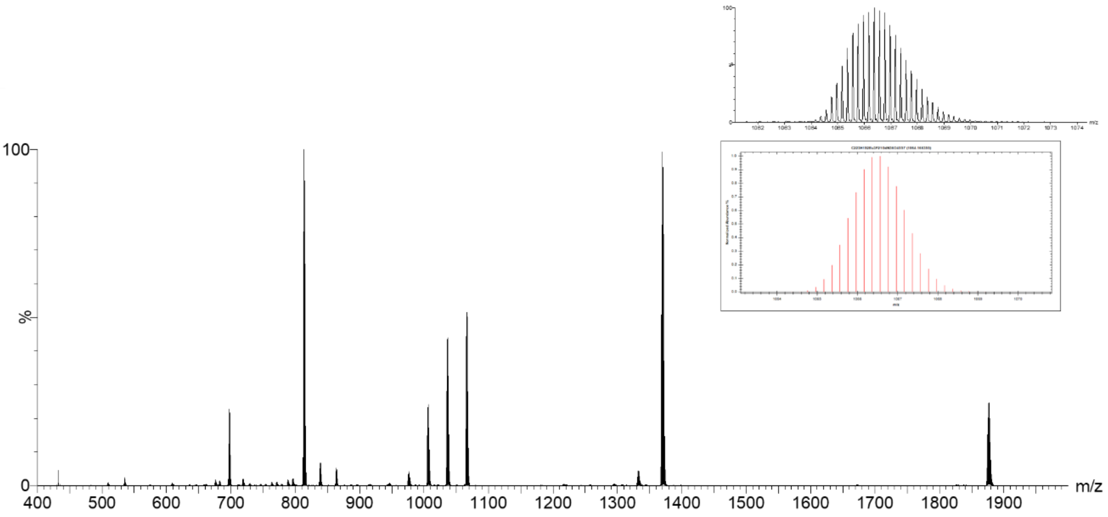

B

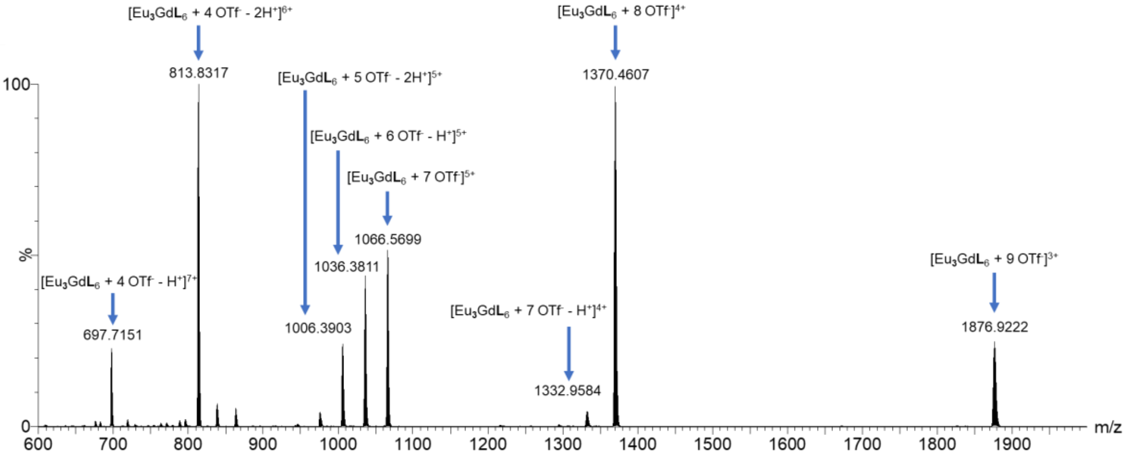

Figure S21. ESI-HRMS of tetrahedra [Eu_nGd_{4-n}**L**$_6$] (n = 0-4, Eu:Gd = 3:1). The chemical formula of the tetrahedra was first confirmed by analyzing the MS peaks with the highest intensity before subjected to MS deconvolution. (A) The full spectrum. Simulated m/z for [Eu_3Gd**L**$_6$ + 7 OTf^-]$^{5+}$ is 1066.5690(100%), Experimental found m/z is 1066.5699(100%). Inset showing the experimental (upper) and calculated(lower) isotopic patterns. (B) Expanded region of the mass spectrum to show the possible assignments of the corresponding prominent peaks.

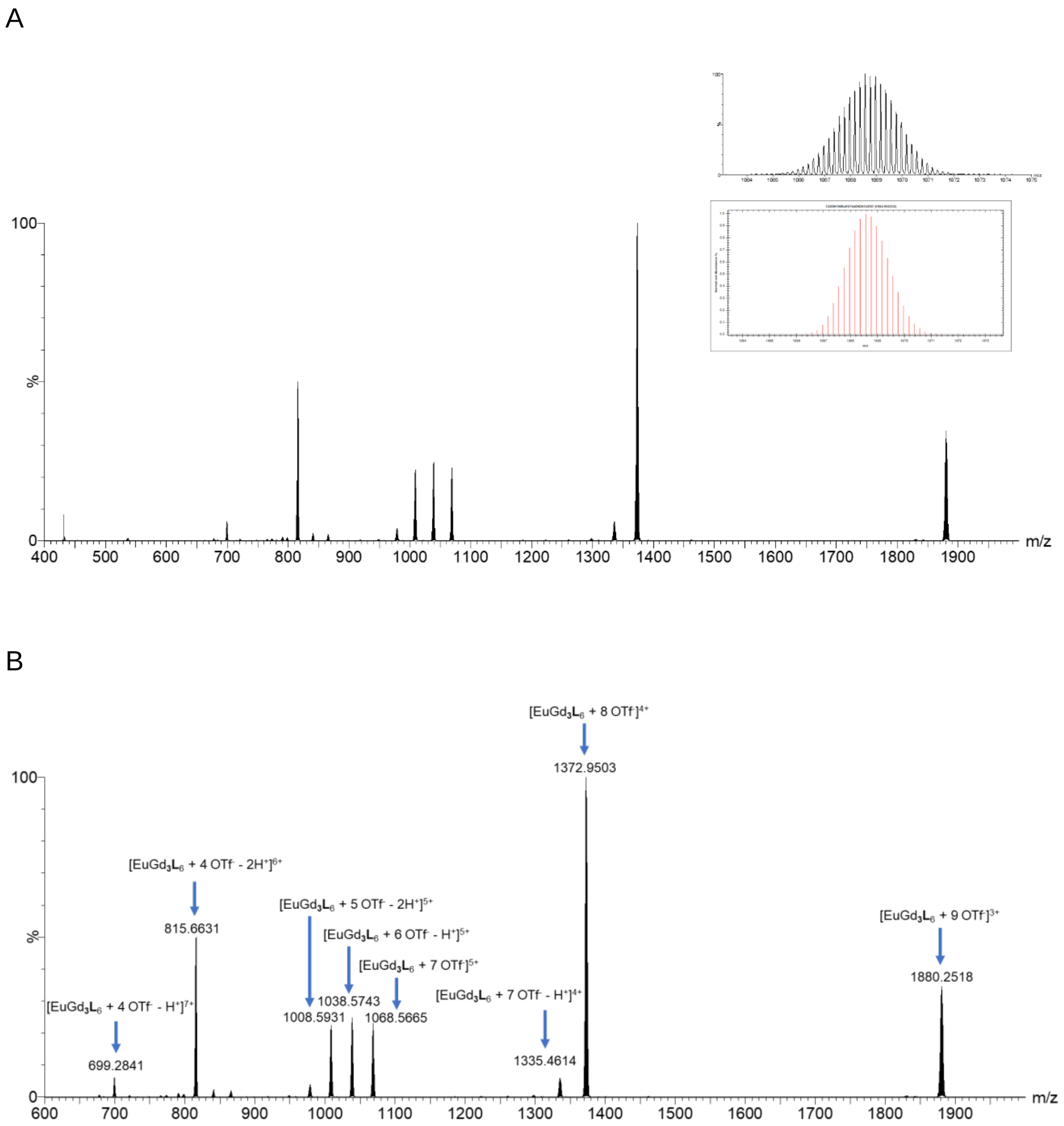

Figure S22. ESI-HRMS of tetrahedra [Eu_nGd_{4-n}**L**$_6$] (n = 0-4, Eu:Gd = 1:3). The chemical formula of the tetrahedra was first confirmed by analyzing the MS peaks with the highest intensity before subjected to MS deconvolution. (A) The full spectrum. Simulated m/z for [$EuGd_3$**L**$_6$ + 7 OTf^-]$^{5+}$ is 1068.5703(100%), Experimental found m/z is 1068.5665(100%). Inset showing the experimental (upper) and calculated(lower) isotopic patterns. (B) Expanded region of the mass spectrum to show the possible assignments of the corresponding prominent peaks.

A

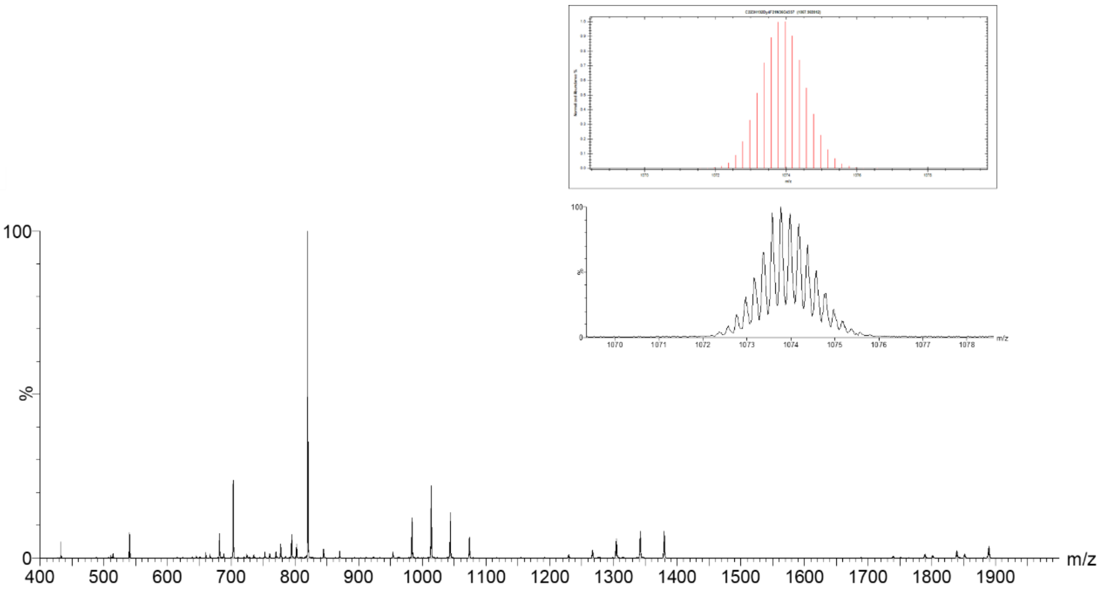

B

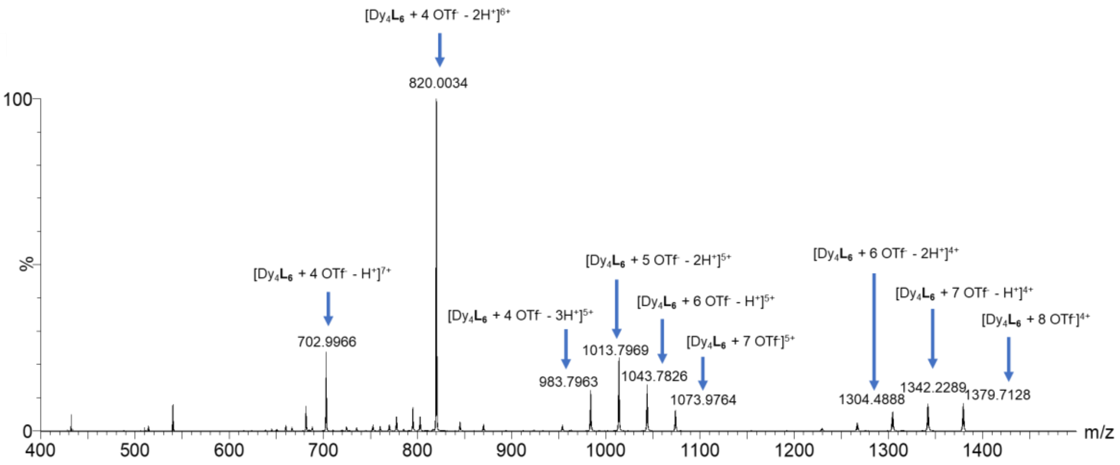

Figure S23. ESI-HRMS of tetrahedron [Dy$_4$**L**$_6$]. (A) The full spectrum. Simulated m/z for [Dy$_4$**L**$_6$ + 7 OTf$^-$]$^{5+}$ is 1073.9743(100%), Experimental found m/z is 1073.9764(100%). Inset showing the experimental (upper) and calculated(lower) isotopic patterns. (B) Expanded region of the mass spectrum to show the possible assignments of the corresponding prominent peaks.

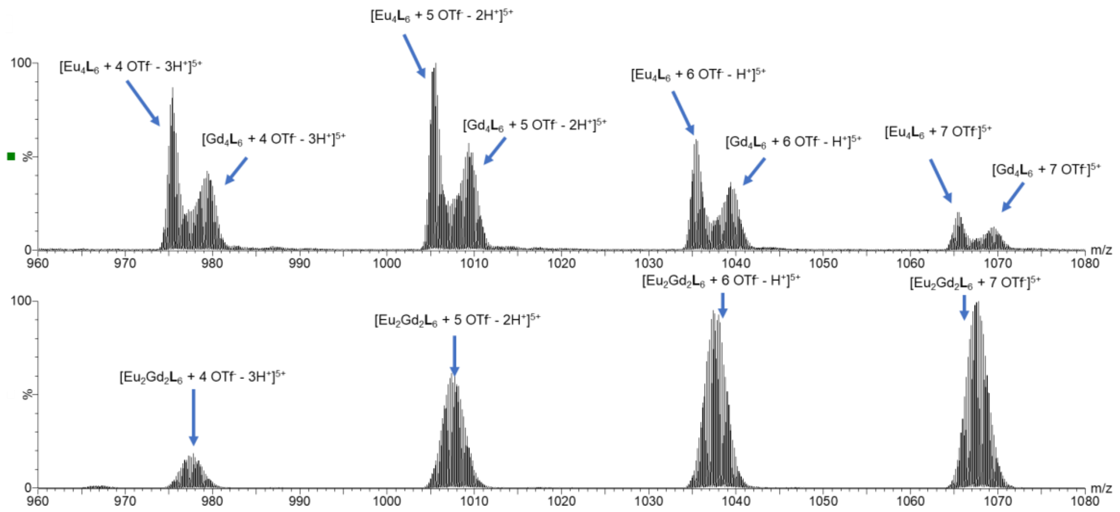

Figure S24. ESI-HRMS of (upper) mixture of [Eu_4**L_6**] and [Gd_4**L_6**] tetrahedron and (bottom) [Eu_nLu_{4-n}**L_6**] (n = 0-4, Eu:Lu = 2:2). The peaks were simply labeled as [Eu_2Gd_2**L_6**] for clearer illustration.

Photophysical measurement

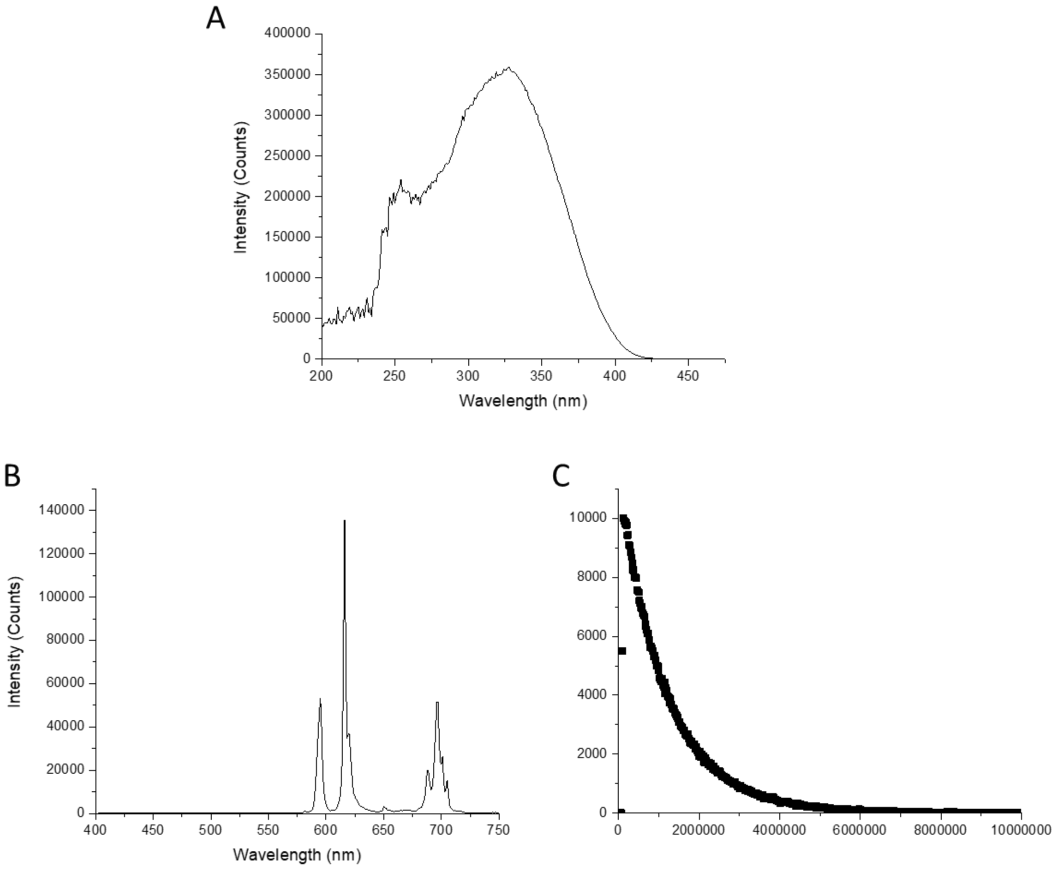

Figure S25. Luminescent data of $[Eu_4\mathbf{L}^{SS}_6]$ (2.50×10^{-6} M in MeCN).(A) Excitation spectrum, λ_{em} = 616 nm, slits = 1.5-1.0, filter 380 nm. (b) Emission spectrum, λ_{ex} = 330 nm, slits = 1.5-1.0, filter 380 nm. (c) Excited state decay curve, λ_{em} = 616 nm, slits = 5-3, filter 380 nm.

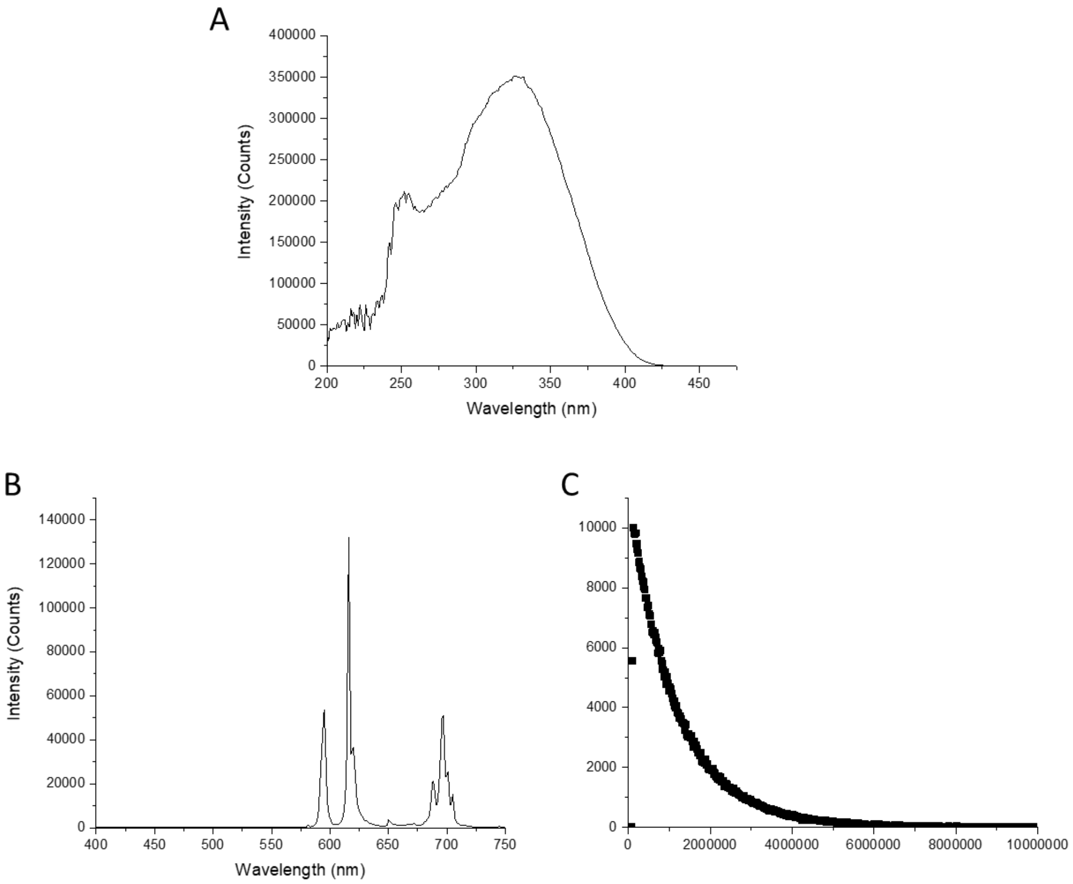

Figure S26. Luminescent data of $[Eu_4\mathbf{L}^{RR}{}_6]$ (2.41 x 10^{-6} M in MeCN). (A) Excitation spectrum, λ_{em} = 616 nm, slits = 1.5-1.0, filter 380 nm. (b) Emission spectrum, λ_{ex} = 330 nm, slits = 1.5-1.0, filter 380 nm. (c) Excited state decay curve, λ_{em} = 616 nm, slits = 5-3, filter 380 nm.

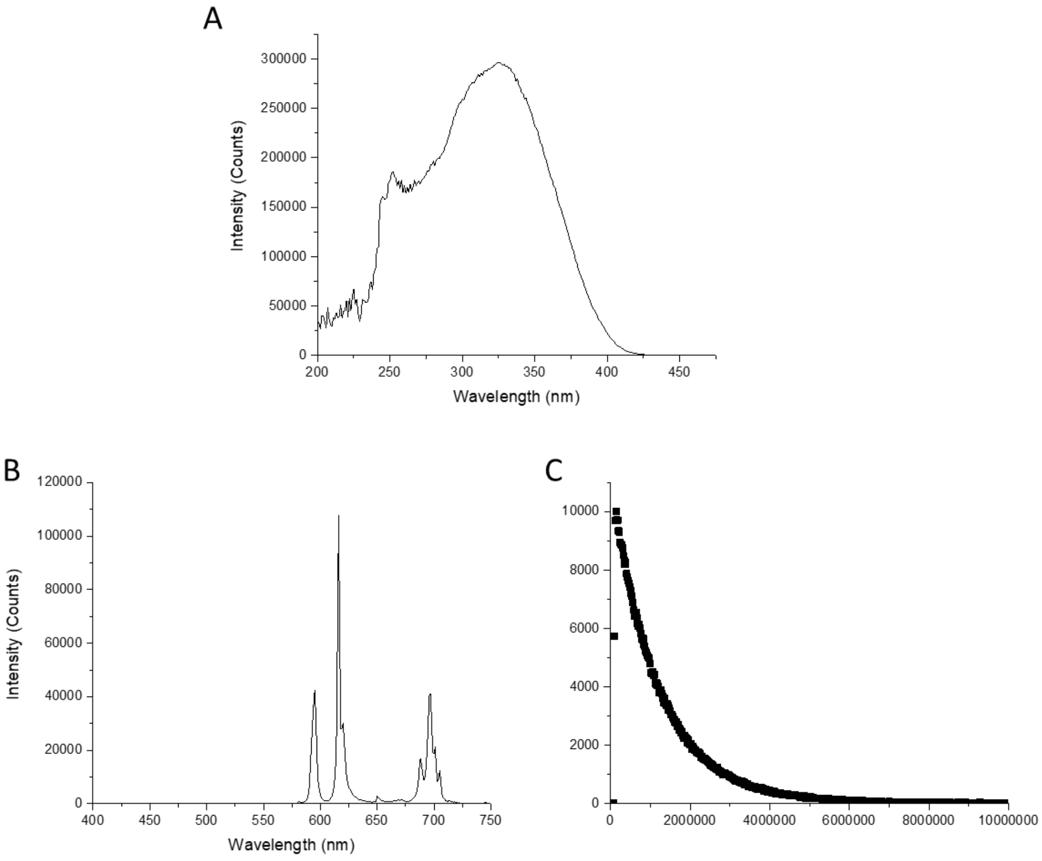

Figure S27. Luminescent data of [$Eu_nGd_{4-n}\mathbf{L}^{SS}_6$] (n = 0-4, Eu:Gd = 3:1) (2.29 x 10^{-6} M in MeCN).(A) Excitation spectrum, λ_{em} = 616 nm, slits = 1.5-1.0, filter 380 nm. (b) Emission spectrum, λ_{ex} = 330 nm, slits = 1.5-1.0, filter 380 nm. (c) Excited state decay curve, λ_{em} = 616 nm, slits = 5.0-3.0, filter 380 nm.

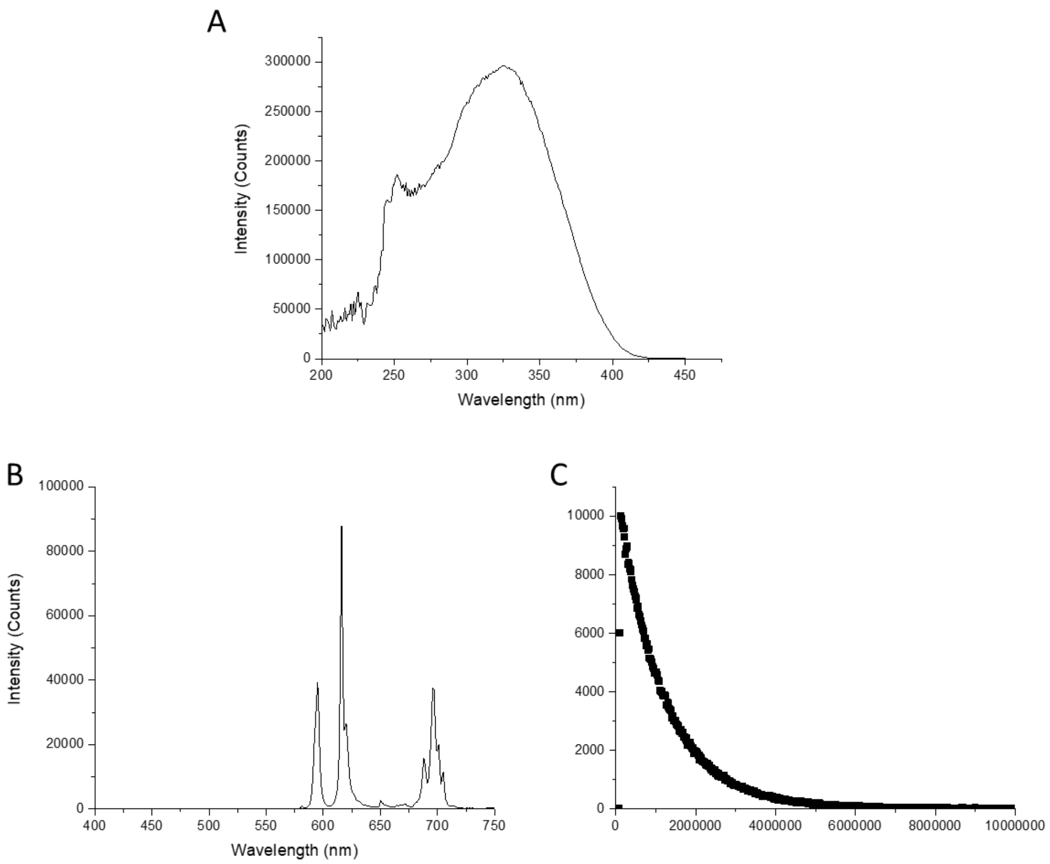

Figure S28. Luminescent data of [Eu_nGd_{4-n}**L**^{RR}$_6$] (n = 0-4, Eu:Gd = 3:1) (2.33 x 10^{-6} M in MeCN).(A) Excitation spectrum, λ_{em} = 616 nm, slits = 1.5-1.0, filter 380 nm. (b) Emission spectrum, λ_{ex} = 330 nm, slits = 1.5-1.0, filter 380 nm. (c) Excited state decay curve, λ_{em} = 616 nm, slits = 5.0-3.5, filter 380 nm.

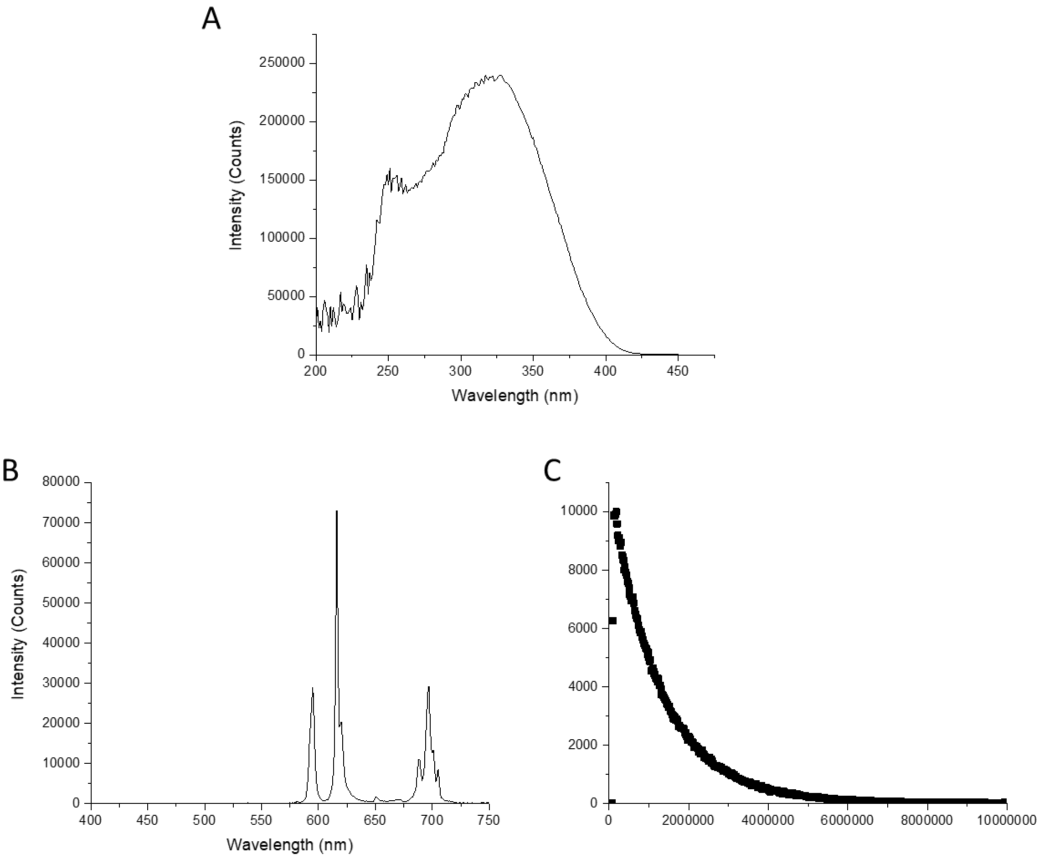

Figure S29. Luminescent data of [Eu_nGd_{4-n}**L**$^{SS}{}_6$] (n = 0-4, Eu:Gd = 1:1) (2.60×10^{-6} M in MeCN).(A) Excitation spectrum, λ_{em} = 616 nm, slits = 1.5-1.0, filter 380 nm. (b) Emission spectrum, λ_{ex} = 330 nm, slits = 1.5-1.0, filter 380 nm. (c) Excited state decay curve, λ_{em} = 616 nm, slits = 5.0-4.0, filter 380 nm.

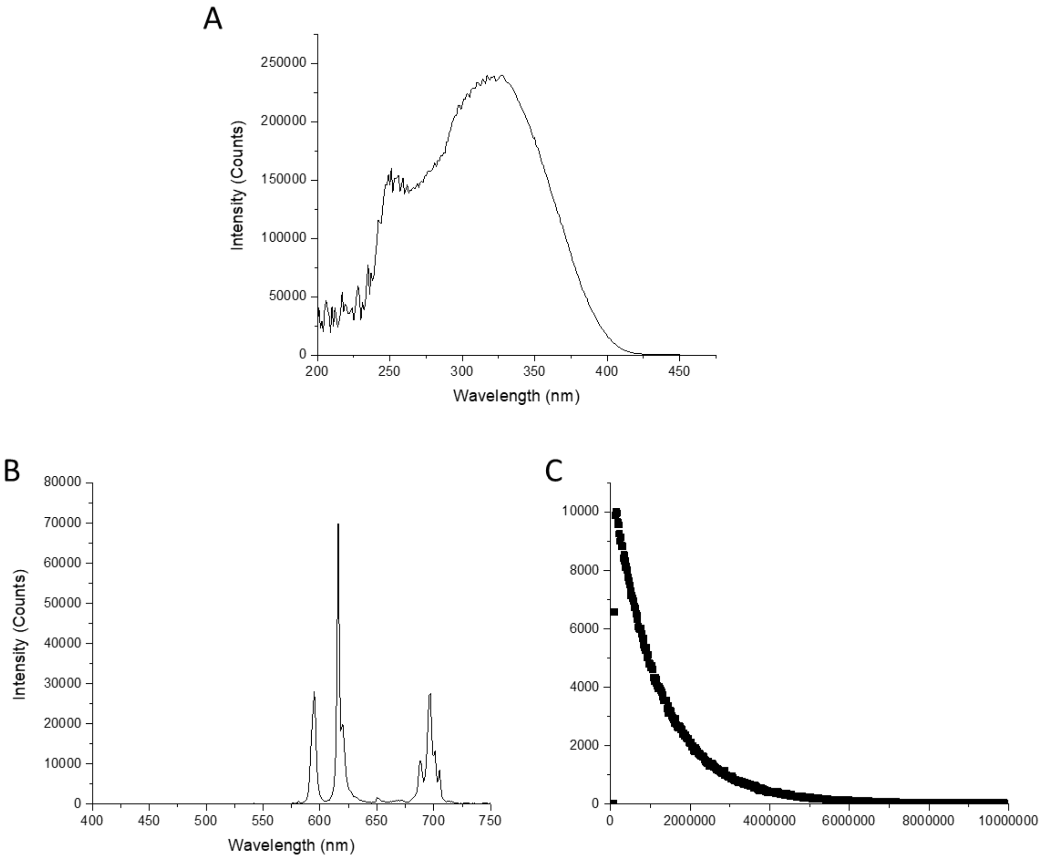

Figure S30. Luminescent data of [Eu_nGd_{4-n}**L**^{RR}$_6$] (n = 0-4, Eu:Gd = 1:1) (2.60 x 10^{-6} M in MeCN).(A) Excitation spectrum, λ_{em} = 616 nm, slits = 1.5-1.0, filter 380 nm. (b) Emission spectrum, λ_{ex} = 330 nm, slits = 1.5-1.0, filter 380 nm. (c) Excited state decay curve, λ_{em} = 616 nm, slits = 5.0-4.0, filter 380 nm.

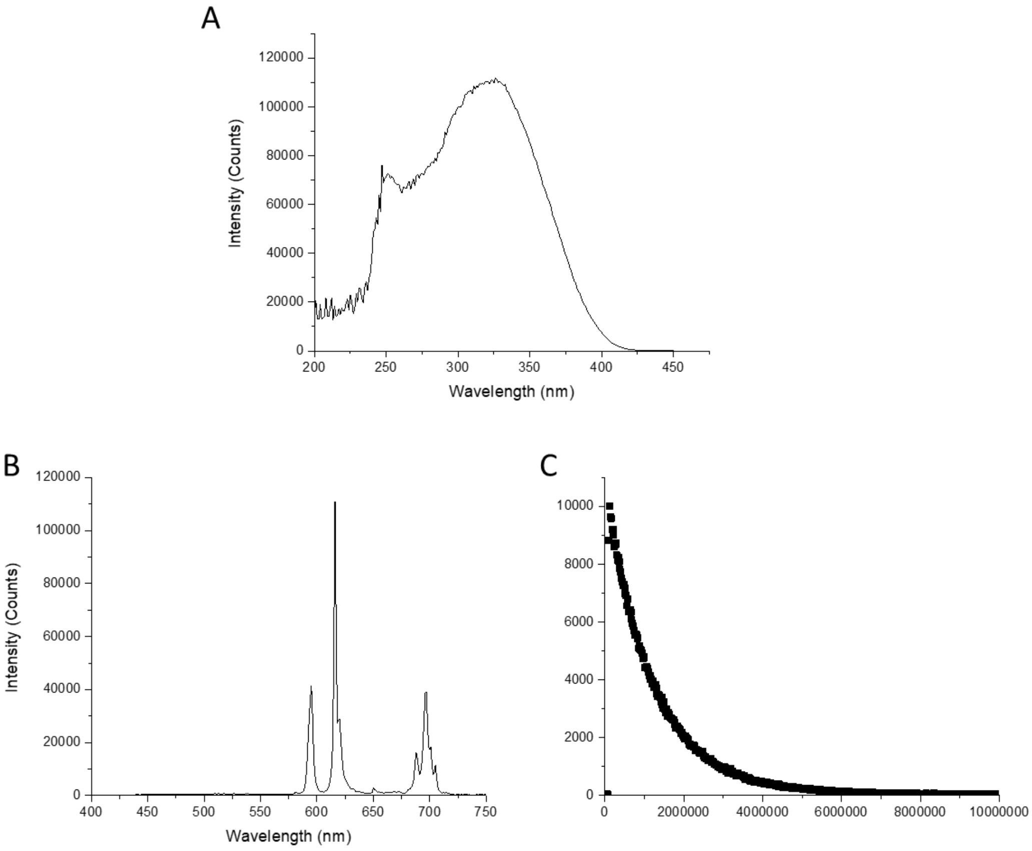

Figure S31. Luminescent data of $[Eu_nGd_{4-n}\mathbf{L}^{SS}{}_6]$ (n = 0-4, Eu:Gd = 1:3) (2.44×10^{-6} M in MeCN).(A) Excitation spectrum, λ_{em} = 616 nm, slits = 2.5-1.0, filter 380 nm. (b) Emission spectrum, λ_{ex} = 330 nm, slits = 2.5-1.0, filter 380 nm. (c) Excited state decay curve, λ_{em} = 616 nm, slits = 6.0-5.0, filter 380 nm.

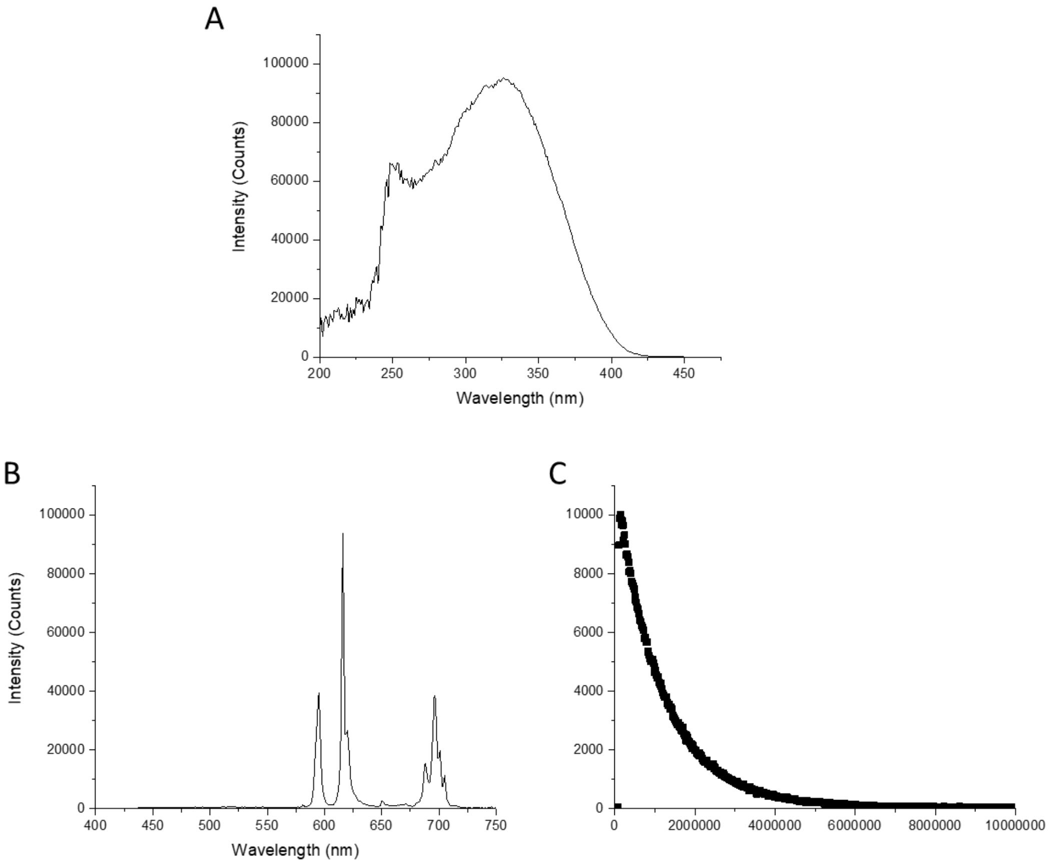

Figure S32. Luminescent data of [Eu_nGd_{4-n}**L**^{RR}$_6$] (n = 0-4, Eu:Gd = 1:3) (2.59 x 10^{-6} M in MeCN).(A) Excitation spectrum, λ_{em} = 616 nm, slits = 2.5-1.0, filter 380 nm. (b) Emission spectrum, λ_{ex} = 330 nm, slits = 2.5-1.0, filter 380 nm. (c) Excited state decay curve, λ_{em} = 616 nm, slits = 6.0-5.0, filter 380 nm.

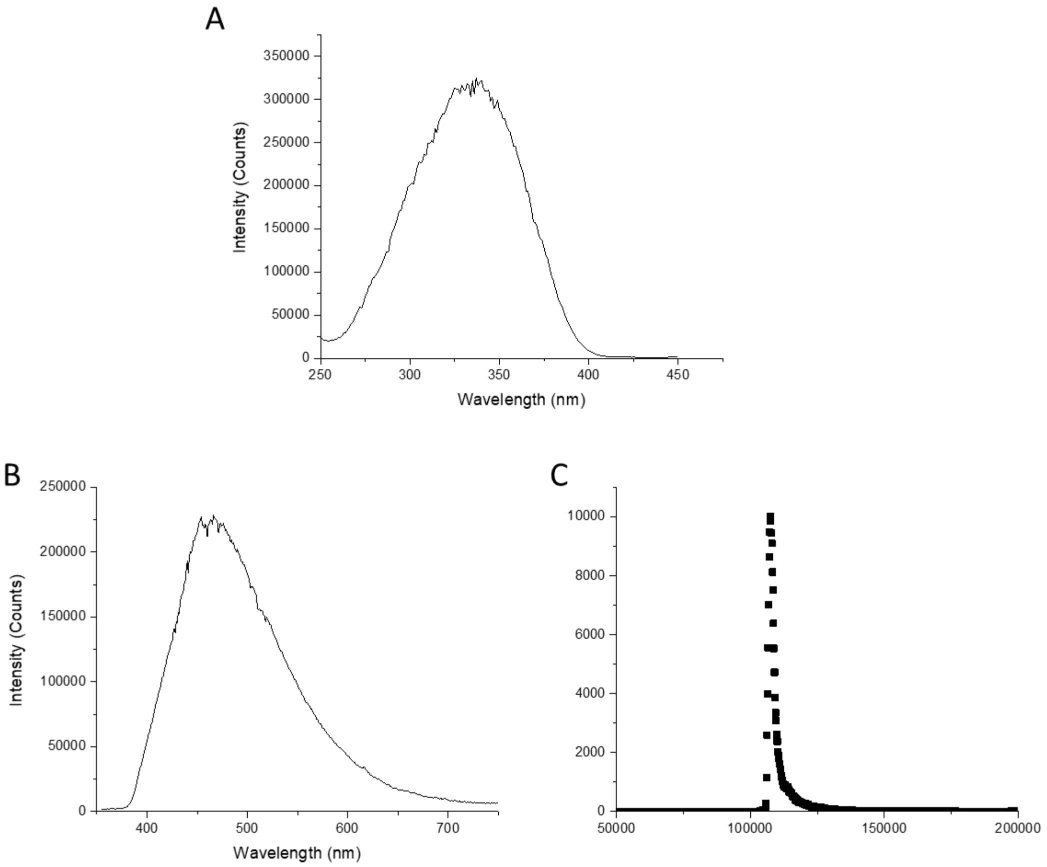

Figure S33. Luminescent data of [Gd_4**L**$^{RR}{}_6$] (9.05 x 10^{-6} M in 1:4 of MeOH/EtOH at 77K).(A) Excitation spectrum, λ_{em} = 466 nm, slits = 2.0-0.5, filter 380 nm. (b) Emission spectrum, λ_{ex} = 330 nm, slits = 2.0-0.5, filter 380 nm. (c) Excited state decay curve, λ_{em} = 466 nm, slits = 2.0-1.0, filter 380 nm.

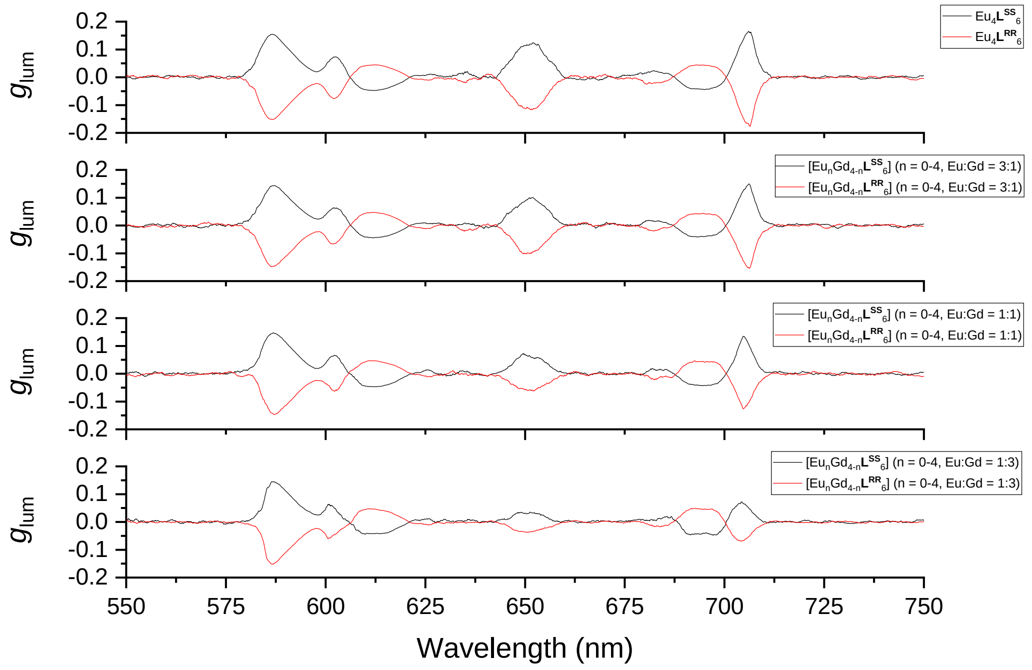

Figure S34. A stack of g_{lum} values of homometallic and heterometallic tetrahedra.

Table S5. Summary of CPL results for the homometallic and heterometallic tetrahedron.

Electronic transition	λ (nm)	g_{lum}							
		[Eu_4**L**$^{SS}{}_6$]	[Eu_4**L**$^{RR}{}_6$]	[Eu_nGd_{4-n}**L**$^{SS}{}_6$] (n = 0-4, Eu:Gd = 3:1)	[Eu_nGd_{4-n}**L**$^{RR}{}_6$] (n = 0-4, Eu:Gd = 3:1)	[Eu_nGd_{4-n}**L**$^{SS}{}_6$] (n = 0-4, Eu:Gd = 1:1)	[Eu_nGd_{4-n}**L**$^{RR}{}_6$] (n = 0-4, Eu:Gd = 1:1)	[Eu_nGd_{4-n}**L**$^{SS}{}_6$] (n = 0-4, Eu:Gd = 1:3)	[Eu_nGd_{4-n}**L**$^{RR}{}_6$] (n = 0-4, Eu:Gd = 1:3)
$^5D_0 \rightarrow {}^7F_1$	587	0.15	-0.15	0.14	-0.15	0.15	-0.15	0.14	-0.15
	601	0.06	-0.06	0.05	-0.06	0.06	-0.05	0.06	-0.06
$^5D_0 \rightarrow {}^7F_2$	613	-0.05	0.04	0.04	-0.05	-0.05	0.05	-0.04	0.04
$^5D_0 \rightarrow {}^7F_3$	652	0.12	-0.11	0.10	-0.10	0.06	-0.06	0.03	-0.04
$^5D_0 \rightarrow {}^7F_4$	685	0.01	-0.01	0.01	-0.01	0.02	-0.01	0.02	-0.01
	697	-0.04	0.04	-0.04	0.04	-0.04	0.04	-0.05	0.05
	704	0.12	-0.12	0.10	-0.11	0.11	-0.10	0.07	-0.07

Table S6. A summary of selected photophysical properties, UV-Vis absorption and luminescence data of lanthanide complexes in acetonitrile solution[a]. [a]using a 10 mm cuvette and filter 380 nm. [b]The relative quantum yields were referenced with quinine sulfate in 0.1 M sulfuric acid (φ= 0.577, λ_{ex} = 350nm) with 10 mm cuvette. [c]Measurement performed at 77K in 1:4 MeOH/EtOH.

	λ_{abs}^{max} (nm)	ε^{max} (L·mol^{-1}·cm^{-1})	λ_{em}^{max} (nm)	φ_x^b (%)	τ (ms)
[Eu_4**L**$^{SS}_6$]	330	121335.7	616	0.81 (0.035)	1.28
[Eu_4**L**$^{RR}_6$]	330	123947.0	616	0.83 (0.035)	1.27
[Eu_nGd_{4-n}**L**$^{SS}_6$] (n = 0-4, Eu:Gd = 3:1)	330	122963.9	616	0.76 (0.03)	1.27
[Eu_nGd_{4-n}**L**$^{RR}_6$] (n = 0-4, Eu:Gd = 3:1)	330	124822.0	616	0.70 (0.025)	1.27
[Eu_nGd_{4-n}**L**$^{SS}_6$] (n = 0-4, Eu:Gd = 1:1)	330	124869.7	616	0.49 (0.025)	1.28
[Eu_nGd_{4-n}**L**$^{RR}_6$] (n = 0-4, Eu:Gd = 1:1)	330	125035.4	616	0.50 (0.02)	1.27
[Eu_nGd_{4-n}**L**$^{SS}_6$] (n = 0-4, Eu:Gd = 1:3)	330	123626.7	616	0.30 (0.02)	1.27
[Eu_nGd_{4-n}**L**$^{RR}_6$] (n = 0-4, Eu:Gd = 1:3)	330	131345.8	616	0.34 (0.03)	1.27
[Gd_4**L**$^{SS}_6$]	330	121829.6	/	/	/
[Gd_4**L**$^{RR}_6$]	330	123657.1	466	/	0.0087[c]

X-ray crystallography data

Crystal structure of [$Eu_nGd_{4-n}L^{SS}_6$] (n = 0-4, Eu:Gd = 1:1):

Single crystals of $C_{225}H_{192}Eu_2F_{27}Gd_2N_{36}O_{51}S_9$ was selected and mounted on a standard Kapton micromount, using neat paratone oil to try and reduce solvent loss during mounting procedure on a Bruker D8 Vantage diffractometer. The crystal was kept at 220.0 K during data collection. Using Olex2[2], the structure was solved with the XT[3] structure solution program using Intrinsic Phasing and refined with the XL[4] refinement package using Least Squares minimisation.

Crystal Data for $C_{224.998875}H_{191.99904}Eu_{1.99999}F_{26.999865}Gd_{1.99999}N_{35.99982}O_{50.999745}S_{8.999955}$ (*M* =5636.07 g/mol): trigonal, space group R3 (no. 146), *a* = 30.0304(9) Å, *c* = 71.671(3) Å, *V* = 55975(4) Å^3, *Z* = 6.00003, *T* = 220.0 K, μ(CuKα) = 5.684 mm^{-1}, *Dcalc* = 1.003 g/cm^3, 70262 reflections measured (5.886° ≤ 2Θ ≤ 117.84°), 34927 unique (R_{int} = 0.0543, R_{sigma} = 0.0620) which were used in all calculations. The final R_1 was 0.0655 (I > 2σ(I)) and wR_2 was 0.1960 (all data). The crystallographic data for the structural analyses have been deposited with the Cambridge Crystallographic Data Centre, CCDC No. 2092789, and the data can be obtained free of charge via www.ccdc.cam.ac.uk/data_request/cif.

Information	Identifier from cif file
Empirical formula	$C_{225}H_{192}Eu_2F_{27}Gd_2N_{36}O_{51}S_9$
Formula weight	5636.07
Temperature/K	220.0
Crystal system	trigonal
Space group	R3
a/Å	30.0304(9)
b/Å	30.0304(9)
c/Å	71.671(3)
α/°	90
β/°	90
γ/°	120
Volume/Å^3	55975(4)
Z	6.00003
ρ_{calc}g/cm^3	1.003
μ/mm^{-1}	5.684
F(000)	17058.0
Crystal size/mm^3	0.23 × 0.18 × 0.13

Radiation	CuKα (λ = 1.54178)
2Θ range for data collection/°	5.886 to 117.84
Index ranges	-33 ≤ h ≤ 33, -33 ≤ k ≤ 33, -79 ≤ l ≤ 79
Reflections collected	70262
Independent reflections	34927 [R_{int} = 0.0543, R_{sigma} = 0.0620]
Data/restraints/parameters	34927/2794/1969
Goodness-of-fit on F^2	0.983
Final R indexes [I>=2σ (I)]	R_1 = 0.0655, wR_2 = 0.1771
Final R indexes [all data]	R_1 = 0.0805, wR_2 = 0.1960
Largest diff. peak/hole / e Å^{-3}	0.80/-0.82
Flack parameter	0.011(3)

Table S7. Results of the Shape Analysis for [Eu_nGd_{4-n}**L**$^{SS}{}_6$] (n = 0-4, Eu:Gd = 1:1).

	Shape measure (°)	
	Tricapped trigonal prism (D_{3h})	Capped square antiprism (C_{4v})
Eu1	5.70	10.30
Eu2	5.70	10.30
Gd3	5.78	10.79
Gd4	5.70	10.30

The coordination geometry of the [Eu_nGd_{4-n}**L**$^{SS}{}_6$] (n = 0-4, Eu:Gd = 1:1) can be best described as tricapped trigonal prism.

References

1. N. Dalla-Favera, J. Hamacek, M. Borkovec, D. Jeannerat, G. Ercolani, C. Piguet, *Inorg. Chem.* **2007**, *46*, 9312-9322

2. O. V. Dolomanov, L. J. Bourhis, R. J. Gildea, J. A. K. Howard, H. Puschmann, *J. Appl. Cryst.* **2009**, *43*, 339-341.

3. G. M. Sheldrick, *Acta Cryst.* **2015**, *A71*, 3-8

4. G. M. Sheldrick, *Acta Cryst.* **2008**, *A64*, 112-122.
